# Multi-source data integration and ensemble machine learning analysis of the role of Corilagin in regulating endoplasmic reticulum stress and alleviating intestinal ischemia reperfusion injury in mice

**DOI:** 10.3389/fphar.2026.1966534

**Published:** 2026-10-09

**Authors:** Zhiyang Wang, Qin Han, Lijie Pang, Siyang Zheng, Xin Wang, Bin Tian, Kun Li, Bin Li, Feng Qiu, Yunpeng Diao, Ruqiang Yuan

**Affiliations:** 1 Institute (College) of Integrative Medicine, Dalian Medical University, Dalian, China; 2 College of Pharmacy, Dalian Medical University, Dalian, China; 3 School of Chemistry and Chemical Engineering, Liaoning Normal University, Dalian, China; 4 College of Advance Institute For Medical Science, Dalian Medical University, Dalian, China

**Keywords:** ABCG2, Corilagin, endoplasmic reticulum stress, intestinal ischemia reperfusion injury, machine learning, NRF2

## Abstract

**Background:**

Intestinal ischemia–reperfusion injury (IIRI) is driven by oxidative stress, excessive inflammation, Endoplasmic Reticulum Stress (ERS), and disruption of the intestinal epithelial barrier. Corilagin, a natural ellagitannin with antioxidant and anti-inflammatory activities, has shown protective effects against IIRI; however, its cross-species and multitarget mechanisms remain unclear.

**Methods:**

This study investigated the protective mechanisms of Corilagin against IIRI by integrating Corilagin-related targets, IIRI-associated genes, and an ERS gene set. Differential expression analyses of GSE37013 and GSE96733 were combined with an ensemble machine-learning framework comprising 15 algorithms to prioritize core genes. Molecular docking and 100-ns all-atom molecular dynamics simulations using the AlphaFold-predicted full-length human NRF2 structure were performed to assess the predicted Corilagin-NRF2 interaction. A mouse IIRI model was used to validate selected molecular and histological findings by histology, RT-qPCR, Western blotting, and immunohistochemistry. In IEC-6 cells subjected to OGD/R, NRF2 immunofluorescence and siRNA-mediated knockdown of Nrf2 or Abcg2 were used to further evaluate the involvement of NRF2 and ABCG2 in Corilagin-mediated protection.

**Results:**

Intersection analysis identified 472 genes shared among Corilagin-related targets, IIRI-related genes, and ERS-associated genes. Enrichment analysis highlighted stress- and injury-related pathways, including PI3K-Akt signaling. Ensemble machine learning prioritized 12 core genes across human and mouse datasets, with ABCG2, IL10, and PRKCZ emerging as prominent candidates. Docking and 100-ns MD simulations supported persistent Corilagin retention within the predicted NRF2 binding region, while post-relaxation MM/GBSA analysis indicated favorable interaction energetics. *In vivo*, Corilagin pretreatment reduced inflammation and oxidative stress-related changes, restored apical ABCG2 and tight-junction protein expression, improved mucosal morphology, and altered CHOP expression. In IEC-6 cells, Nrf2 or Abcg2 knockdown weakened Corilagin-mediated preservation of Occludin, Claudin-1, and ZO-1 under OGD/R, supporting NRF2–ABCG2 involvement in this protective response.

**Conclusion:**

Corilagin may alleviate IIRI by modulating ERS-related molecular changes and preserving the intestinal epithelial barrier with NRF2 and ABCG2 contributing to a candidate regulatory module involved in this protective response. These findings provide computational and experimental support for further investigation of Corilagin as a potential intervention for IIRI and illustrate the value of combining multi-source data integration with ensemble machine learning to characterize the multitarget pharmacology of natural products.

## 1 Introduction

IIRI is a critical emergency with a high mortality rate in surgical practice. It commonly occurs in mesenteric vascular embolism, severe trauma, hemorrhagic shock, and major abdominal surgery (Deng et al., 2026; Bala et al., 2022). Restoration of blood flow after transient intestinal ischemia triggers a cascade of pathological injury, including a sharp increase in oxidative stress, extensive release of inflammatory factors, and disruption of intestinal epithelial tight junctions (Chu et al., 2023; Wang et al., 2025). These changes cause intestinal mucosal necrosis and loss of intestinal absorptive and barrier functions (Zhang et al., 2023a; Deng et al., 2022). They also induce bacterial and endotoxin translocation and systemic inflammatory response syndrome, which further causes distant organ injury such as acute lung injury and multiple organ failure and markedly increases the risk of death (Tang et al., 2025; Deng et al., 2023). Current management of IIRI relies on timely diagnosis, restoration of perfusion when feasible, surgery or endovascular intervention, and supportive care; no specific pharmacological therapy directly prevents reperfusion injury (Theodore et al., 2024; Zhang et al., 2023b; M et al., 2023). Therefore, a major research direction in the field of intestinal injury is to identify natural bioactive substances with low toxicity and high efficacy and systematically clarify their molecular regulatory networks in IIRI.

ERS is a key process that mediates the pathological progression of IIRI. Disruption of the oxidative microenvironment caused by ischemia reperfusion leads to the accumulation of large amounts of misfolded proteins in the endoplasmic reticulum and activates the adaptive unfolded protein response (Shi et al., 2024). If stress remains excessive, the endoplasmic reticulum homeostatic regulatory pathways that initially have protective effects shift to a pro-apoptotic program, accelerating intestinal epithelial cell death and aggravating intestinal barrier disruption and local inflammatory infiltration (Zhao et al., 2024). Existing studies have shown that several signaling pathways interact with ERS and jointly promote the progression of IIRI. Dysregulated PI3K–Akt signaling, ferroptotic lipid peroxidation, and HMGB1–RAGE-associated inflammatory signaling have each been implicated in IIRI (Zou and Wang, 2022; Mi et al., 2021). These pathways interact to form a highly complex pathological regulatory network. However, current mechanistic studies have clear limitations. Most have only performed fragmented validation of a single gene or pathway (Alicehajic et al., 2024) and lack global molecular network analyses across humans and mice and across multiple omics dimensions (Kang et al., 2022; Zong et al., 2023). This makes it difficult to systematically identify the key molecules that regulate the pathological progression of IIRI.

Corilagin is a natural bioactive ellagitannin derived from traditional medicinal plants such as Terminalia chebula, Phyllanthus urinaria, and Dimocarpus longan (Wang et al., 2023a). It has been reported to show a relatively favorable safety profile and multitarget pharmacological properties, including antioxidant, anti-inflammatory, anti-apoptotic, intestinal-protective, hepatoprotective, and other biological activities (Wang et al., 2023a). A few preliminary studies have shown that Corilagin can alleviate intestinal injury caused by IIRI in mice and that its intestinal-protective effect is associated with the regulation of ERS. For example, it can inhibit the NLRP3 inflammasome and pyroptosis and simultaneously reduce primary intestinal injury and secondary lung injury (Li et al., 2022). It can activate the AMPK/Sirt1 autophagy pathway and alleviate oxidative stress and apoptosis in the intestinal epithelium (Li et al., 2023). It can also bind to the endoplasmic reticulum chaperone BiP and attenuate excessive ERS (Wang et al., 2024a). Separately, it can inhibit NCOA4-mediated ferroptosis (Wang et al., 2023b). However, these studies have clear limitations. The studies are fragmented and focus only on validating a single pathway. They have not systematically identified all potential targets through which Corilagin acts on IIRI and regulates ERS, and they also lack cross-validation using human and mouse data. They therefore cannot fully explain its overall multitarget pharmacological profile. In addition, natural products often exhibit polypharmacology across multiple targets and pathways. Conventional experimental approaches based on a single gene or pathway make it difficult to comprehensively capture the interactions between natural compounds and complex disease molecular networks, which substantially limits in-depth investigation of the intestinal-protective mechanism of Corilagin.

A computational strategy combining multi-source data integration and ensemble machine learning provides a new approach for analyzing candidate regulatory networks in complex diseases and natural medicines (Saldívar-González et al., 2022; Zhu et al., 2022). This strategy can integrate target information from multiple databases with high-throughput cross-species transcriptomic data (Rao et al., 2023; Hansen et al., 2019). Multi-algorithm ensemble models can prioritize core genes from heterogeneous data and help characterize candidate regulatory networks of natural bioactive compounds (Shokhirev and Johnson, 2022). Based on this background and the gaps in existing research, this study established an integrated research framework. Corilagin targets predicted from multiple databases, IIRI-related genes, and an ERS gene set were integrated for preliminary intersection analysis. Differential expression analysis was performed using human and mouse GEO transcriptomic datasets, and an ensemble machine learning framework comprising 15 algorithms was used to prioritize genes across the analyzed datasets. Molecular simulation was used to analyze the predicted interaction between the bioactive molecule and a key transcriptional protein. Finally, *in vivo* and *in vitro* models were established for experimental validation, and selected molecular, cellular, and histopathological outcomes were assessed using histology, RT-qPCR, Western blotting, and immunohistochemistry. This study aimed to identify core genes associated with the effects of Corilagin on IIRI and ERS and to analyze the multitarget characteristics of Corilagin in IIRI and to provide candidate targets and a preliminary experimental and theoretical basis for further studies of natural medicines in IIRI.

## 2 Materials and methods

### 2.1 Study design and technical workflow

This study integrated bioinformatics analysis, machine learning, molecular simulation, and animal experiments. First, potential Corilagin targets, genes related to IIRI, and genes related to ERS were collected to construct a candidate target set. Public transcriptomic data were then preprocessed and analyzed for differential expression, and core genes were identified using functional enrichment, a protein-protein interaction (PPI) network, and machine learning. Molecular docking and molecular dynamics simulations were further used to evaluate the predicted binding pose and simulated stability of Corilagin with NRF2. Subsequently, a mouse IIRI model was established to examine the expression of core genes, intestinal mucosal injury, ABCG2 expression, ERS-related changes, and barrier-associated proteins using RT-qPCR, Western blotting, hematoxylin and eosin (H&E) staining, and immunohistochemistry (IHC). Finally, an IEC-6 OGD/R model was used for *in vitro* mechanistic evaluation, including NRF2 immunofluorescence and siRNA-mediated knockdown of Nrf2 or Abcg2 to assess their involvement in Corilagin-mediated preservation of tight-junction proteins (Figure 1).

**FIGURE 1:**
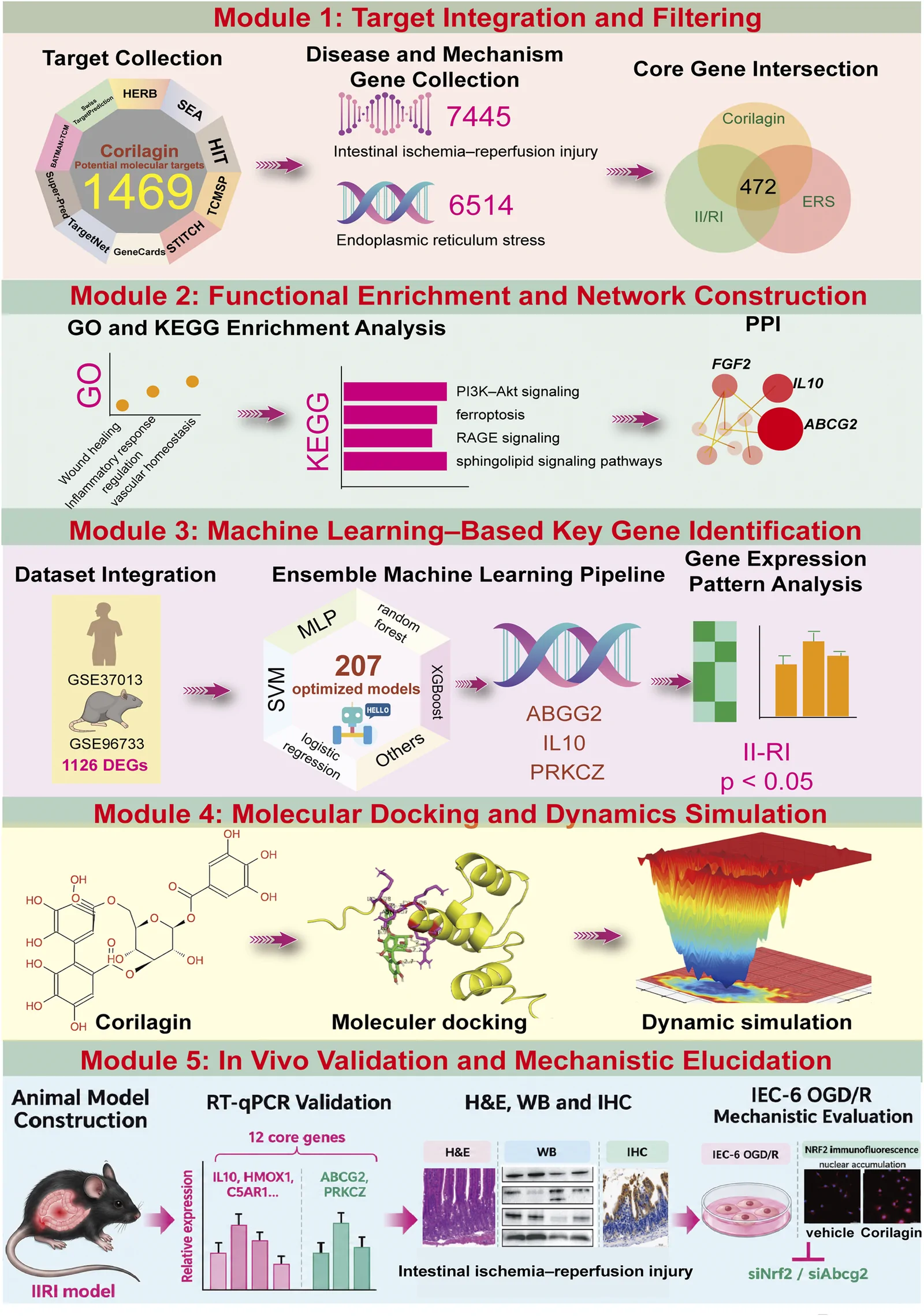
Overview of the complete study workflow. Module 1: Potential Corilagin targets were collected from multiple databases, such as HERB, SEA, and SwissTargetPrediction, and intersected with IIRI- and ERS-related genes to obtain 472 overlapping genes. Module 2: GO and KEGG enrichment analyses linked these genes to PI3K-Akt signaling, and other stress-related pathways. Protein–protein interaction (PPI) network analysis identified ABCG2 and IL10 as candidate central nodes. Module 3: Public transcriptomic datasets GSE37013 and GSE96733 were analyzed. An ensemble consisting of 15 machine-learning models, such as SVM, MLP, and random forest, prioritized 12 core genes, among which ABCG2, IL10, and PRKCZ were prominent. Module 4: Molecular docking and molecular dynamics simulations were used to characterize the predicted Corilagin–NRF2 interaction. Despite substantial global conformational rearrangement of NRF2 during the simulation, Corilagin remained associated with the predicted binding region, and MM/GBSA analysis of the post-relaxation trajectory segment indicated favorable end-state interaction energetics. Module 5: Experimental validation was performed using a mouse IIRI model and an IEC-6 OGD/R model. *In vivo*, Corilagin increased ABCG2 expression, modulated the ERS-related marker CHOP, and preserved intestinal mucosal morphology and tight-junction protein expression. *In vitro*, Corilagin promoted NRF2 nuclear accumulation, while Nrf2 or Abcg2 knockdown attenuated its protective effects on Occludin, Claudin-1, and ZO-1.

### 2.2 Target acquisition and integration

Corilagin-related targets were obtained from databases including SwissTargetPrediction, BATMAN-TCM, GeneCards, HERB, HIT, HTDocking, SEA, STITCH, SuperPred, TargetNet, and TCMSP. After the results from all databases were combined and duplicate genes were removed, 1,469 potential Corilagin targets were retained (Supplementary Material 1). IIRI-related genes were retrieved from the GeneCards, GSEA, and OMIM databases using the keyword “intestinal ischemia reperfusion injury/IIRI”, and 7445 IIRI-related genes were obtained (Supplementary Material 2). ERS-related genes were collected from the GeneCards and GSEA databases, yielding 6514 ERS-related genes (Supplementary Material 3).

The intersection of the Corilagin targets, IIRI-related genes, and ERS-related genes yielded 472 shared genes (Supplementary Material 4), which were used for subsequent functional enrichment analysis and network construction.

### 2.3 Acquisition and preprocessing of human and mouse IIRI microarray data

The human GSE37013 and mouse GSE96733 IIRI microarray datasets were downloaded from the Gene Expression Omnibus (GEO) database. GSE37013 corresponded to platform GPL6947, and GSE96733 corresponded to platform GPL23038. Probe IDs were converted to official gene symbols using the platform annotation files. When multiple probes corresponded to the same gene, their mean expression value was used. Missing values were imputed using the impute. knn function, duplicate probes were merged using the avereps function, and genes with a mean expression value below 0.7 were removed (Shokhirev and Johnson, 2022; Clough et al., 2024; Wratten et al., 2021). The normalize Between Arrays function was then used for between-sample normalization, and the expression matrix was log2-transformed (Ritchie et al., 2015).

The groups for differential expression analysis were as follows. In the human dataset, the 30 min reperfusion group was compared with the control group, the 120 min reperfusion group was compared with the control group, and the combined group of the two reperfusion time points was compared with the control group. In the mouse dataset, the 3 h reperfusion group was compared with the control group, the 6 h reperfusion group was compared with the control group, and the combined group of the two reperfusion time points was compared with the control group. Considering that FDR (Benjamini–Hochberg) correction in the preliminary analysis resulted in too few differentially expressed genes to support subsequent intersection analysis, functional enrichment, and ensemble machine-learning analyses, differential expression analysis was performed using the limma package in R, with thresholds of |log2 fold change| ≥ 1 and *P* < 0.05 (Ritchie et al., 2015). Mouse genes were converted to human homologous genes using HomoloGene, and the differential expression results from the human and mouse datasets were integrated.

### 2.4 GO and KEGG enrichment analyses

Gene Ontology (GO) and Kyoto Encyclopedia of Genes and Genomes (KEGG) enrichment analyses were performed using clusterProfiler 4.6.0 in R. GO analysis included three categories: biological process, cellular component, and molecular function (Wu et al., 2021; Zhao and Rhee, 2023). GO database release 202,504 and KEGG database release 104.1 (2025–04) were used as annotation sources, and the whole genome was used as the background set for the hypergeometric test. Multiple testing was corrected using the Benjamini–Hochberg method, and terms with a *P* < 0.05 were considered significantly enriched (Wu et al., 2021; Zhao and Rhee, 2023; Benjamini and Hochberg, 1995).

### 2.5 PPI network construction and key gene selection

Key genes were converted to Entrez Gene IDs and submitted to the STRING v12.0 database. The species was restricted to humans (*Homo sapiens*, taxid: 9,606), and the minimum interaction confidence was set to 0.7. After isolated nodes without interactions were removed, the network was imported into Cytoscape 3.9.1 and arranged using the ForceAtlas2 algorithm. CytoNCA 2.5.0 was used to calculate degree, betweenness centrality, and closeness centrality. Nodes ranked in the top 10% of the network for all three topological parameters were considered potential key nodes.

### 2.6 Machine learning and core gene selection

Probe ID-gene symbol conversion and log2 transformation were completed for the GSE37013 and GSE96733 datasets. Genes shared across the datasets were extracted and intersected with 73 candidate targets to obtain the expression matrix for machine learning. Feature selection was performed using LASSO regression, and the variables corresponding to lambda. min were selected as candidate features. In the R 4.4.1 environment, packages including keras, xgboost, randomForest, gbm, and e1071 were used to construct 15 binary classification models: neural network, logistic regression, discriminant analysis, k-nearest neighbors, decision tree, random forest, XGBoost, ridge, LASSO, elastic net, support vector machine, gradient boosting machine, backpropagation neural network, stepwise logistic regression, and naive Bayes (Greener et al., 2022; Collins et al., 2024).

Hyperparameters, including learning rate, dropout, alpha, mtry, and kernel function type, were optimized by grid search, and five-fold cross-validation was performed using the caret package (Greener et al., 2022; Collins et al., 2024). Model performance at three probability thresholds was evaluated using F1 score, accuracy, recall, and area under the receiver operating characteristic curve (AUC), and models with a mean evaluation metric greater than 0.5 were retained (Shokhirev and Johnson, 2022). Genes were comprehensively ranked based on their selection frequency and feature importance across the models, and high-frequency core genes were selected for subsequent experimental validation.

### 2.7 Molecular docking

The structure of human NRF2 (AlphaFold model AF-Q16236-F1) was obtained from the AlphaFold Protein Structure Database and used as the receptor, while Corilagin (PubChem CID: 73568) served as the ligand. The receptor was standardized and its protonation state and hydrogen-bonding network were optimized at pH 7.4 using PROPKA. Corilagin was converted from 2D to 3D using OpenBabel and subjected to 500 steps of conjugate-gradient energy minimization with the MMFF94s force field. Gasteiger charges were assigned using OpenBabel v3.1.1, polar hydrogens were retained for the receptor, and ligand rotatable bonds were defined before conversion to PDBQT format. Molecular docking was performed using AutoDock Vina with an exhaustiveness of 256, num_modes = 10, and energy_range = 3 kcal/mol. The ligand was treated as flexible and the receptor as rigid, and the pose with the lowest predicted binding affinity was selected for subsequent interaction analysis and molecular dynamics simulation (Eberhardt et al., 2021; Paggi et al., 2024).

### 2.8 Molecular dynamics simulation and MM/GBSA binding-energy estimation

Molecular dynamics (MD) simulations were performed using the GROMACS 2025.1 software package. The system was parameterized using the AMBER99SB-ILDN force field, and the SPC/E water model was selected for explicit solvation. To ensure a physically sound periodic boundary condition, the total charge of the system was neutralized by adding an appropriate number of Na^+^ and Cl^−^ counterions. All simulations were conducted at a constant temperature of 300 K and a standard pressure of 1.0 bar. The simulation protocol was initiated with an energy minimization of the position-restrained complex using the steepest descent algorithm for a maximum of 50,000 steps to eliminate initial steric clashes. Subsequently, the system was equilibrated in two successive phases: an isothermal-isochoric (NVT) ensemble and an isothermal-isobaric (NPT) ensemble. Both equilibration phases were conducted for 100 ps (50,000 steps) with a time step of 2 fs? The temperature was regulated using the v-rescale thermostat with a coupling time constant of 0.1 ps, while the pressure was maintained using the Parrinello-Rahman barostat. Following equilibration, an unrestrained production MD simulation was executed for 100 ns (50, 000, 000 steps) with a time step of 2 fs? Throughout the simulation, the LINCS algorithm was applied to constrain all bonds involving hydrogen atoms. Long-range electrostatic interactions were calculated using the Particle Mesh Ewald (PME) method, with the short-range cutoff radius set to 1.0 nm. After the simulation was completed, the built-in tool of the software was usedto analyze the trajectory, and the root-mean-square variance (RMSD), root-meansquare fluctuation (RMSF) and protein rotation radius of each amino acid trajectorywere calculated, combined with the free energy topography and other data. MM/GBSA calculations were performed using gmx_MMPBSA. Based on the RMSD profile, the equilibrated 60–100 ns segment of the production trajectory was used for the binding-energy analysis, and 401 snapshots were uniformly extracted from this interval. The MM/GBSA energy estimate was calculated as ΔGMM/GBSA = Gcomplex − Gprotein − Gligand and decomposed into van der Waals, electrostatic, polar-solvation, and nonpolar-solvation contributions. The entropic contribution was additionally evaluated using the interaction entropy (I.E.,) method at 300 K. The reliability of the, I.E., estimate was assessed from the standard deviation of the interaction energy, with values exceeding approximately 3.6 kcal/mol considered insufficiently converged for quantitative interpretation (Valdés-Tresanco et al., 2021).

### 2.9 Experimental animals and the IIRI model

6-week-old male C57BL/6 J mice were purchased from the Laboratory Animal Center of Dalian Medical University. All animal experiments were approved by the Animal Care and Ethics Committee of Dalian Medical University (AEE19007, 2021-6-2) and strictly followed the relevant regulations for laboratory animal management. Food was withheld for 12 h before surgery, with free access to water. After the mice were anesthetized with tribromoethanol (150 mg/kg), the superior mesenteric artery (SMA) was occluded for 45 min, followed by restoration of blood flow and reperfusion for 1 h to establish the IIRI model (Li et al., 2022; Wang et al., 2024a; Wang et al., 2023b).

Mice were randomly divided into four groups, with 10 mice per group. From each group, three mice were randomly assigned to Western blotting and RT-qPCR, five mice were assigned to H&E staining and IHC, and the remaining two mice were reserved. Mice in the sham + vehicle group (control group) received a daily intraperitoneal injection of saline for 7 days, followed by sham surgery. Mice in the IIRI + vehicle group (model group) were pretreated with saline for 7 days and then subjected to IIRI. Mice in the sham + Corilagin group (Corilagin-only group) received intraperitoneal Corilagin (CAS No. 23094–69-1; Cat. No. A10085; purity 99.7%) at 30 mg/kg once daily for seven consecutive days, followed by sham surgery. The dose was selected with reference to a previously published dose-escalation study using the same IIRI model (Wang et al., 2023b). Mice in the IIRI + Corilagin group (treatment group) received the same dose of Corilagin as pretreatment and were then subjected to IIRI.

### 2.10 OGD/R model and siRNA-mediated gene knockdown

Rat small intestinal crypt epithelial IEC-6 cells were cultured in Dulbecco’s modified Eagle’s medium (DMEM) supplemented with 10% fetal bovine serum, 50 U/mL penicillin, and 50 U/mL streptomycin at 37 °C in a humidified atmosphere containing 5% CO_2_. Cells in the logarithmic growth phase were used for subsequent experiments. To mimic IIRI *in vitro*, an OGD/R model was established as previously described (Li et al., 2017). Briefly, the complete culture medium was replaced with serum- and glucose-free DMEM, and the cells were incubated at 37 °C in a hypoxic chamber containing 1% O_2_, 5% CO_2_, and 94% N_2_ for 1 h. The cells were then returned to complete DMEM under normoxic conditions for 1 h to simulate reperfusion.

For gene knockdown, IEC-6 cells were transfected with small interfering RNAs (siRNAs) targeting rat Nrf2 or Abcg2 using Lipofectamine™ 3,000 Transfection Reagent (Invitrogen, Thermo Fisher Scientific, Waltham, MA, USA) according to the manufacturer’s instructions. Briefly, siRNAs were diluted in Opti-MEM^TM^ I Reduced Serum Medium and transfected at a final concentration of 50 nM. After 6 h of transfection, the medium was replaced with complete culture medium, and the cells were cultured for an additional 42 h before subsequent Corilagin and OGD/R treatments. The siRNA sequences were as follows: siNC sense, 5′-UUC​UCC​GAA​CGU​GUC​ACG​UTT-3′, and antisense, 5′-ACG​UGA​CAC​GUU​CGG​AGA​ATT-3′; siNrf2 sense, 5′-GCA​UGC​UAC​GUG​AUG​AAG​ATT-3′, and antisense, 5′-UCU​UCA​UCA​CGU​AGC​AUG​CTT-3′; siAbcg2 sense, 5′-GGA​UGA​GGU​UGU​UUA​UAG​UTT-3′, and antisense, 5′-ACU​AUA​AAC​AAC​CUC​AUC​CTT-3′.

IEC-6 cells were divided into five groups: siNC, siNC + OGD/R, siNC + OGD/R + Corilagin, siNrf2 + OGD/R + Corilagin, and siAbcg2 + OGD/R + Corilagin. Cells in the siNC group were transfected with negative-control siRNA and maintained under normoxic conditions. Cells in the siNC + OGD/R group were transfected with siNC and subsequently subjected to OGD/R. Cells in the siNC + OGD/R + Corilagin group were transfected with siNC and treated with Corilagin for 24 h before OGD/R exposure. For gene-knockdown experiments, cells were transfected with siNrf2 or siAbcg2 for 48 h and subsequently treated with Corilagin and subjected to OGD/R under the same conditions. Following treatment, the expression levels of Occludin, Claudin-1, and ZO-1 were determined by Western blotting.

### 2.11 Cell viability assessment using the CCK-8 assay

The effects of Corilagin on IEC-6 cell viability under basal culture conditions were evaluated using a CCK-8 Cell Proliferation And Cytotoxicity Assay Kit (Solarbio, China, CA1210). IEC-6 cells were seeded in 96-well plates at a density of 5 × 10^3^ cells per well in 100 μL of complete culture medium and incubated for 24 h at 37 °C in a humidified atmosphere containing 5% CO_2_. The medium was then replaced with 100 μL of fresh complete medium containing Corilagin at final concentrations of 0, 0.25, 0.5, 0.75, 1, 1.25, 1.5, 1.75 or 2 μM, and the cells were incubated for an additional 24 h under normoxic conditions without oxygen–glucose deprivation. Corilagin was dissolved in dimethyl sulfoxide (DMSO) before dilution in complete medium, and the final DMSO concentration was maintained at 0.1% across all treatment conditions.

At the end of treatment, 10 μL of CCK-8 reagent was added directly to each well, and the plate was incubated at 37 °C for 1 h, protected from light. Absorbance was measured at 450 nm using a microplate reader. Cell-free wells containing the same medium, corresponding concentration of Corilagin or vehicle, and CCK-8 reagent were processed in parallel as concentration-matched blanks to account for noncellular background absorbance and potential compound–reagent interference. Relative cell viability was calculated as follows: cell viability (%) = [(A_treatment − A_blank, treatment)/(A_vehicle − A_blank, vehicle)] × 100, where A_treatment and A_vehicle represent the absorbance values of Corilagin-treated and vehicle-treated cells, respectively, and A_blank, treatment and A_blank, vehicle represent their corresponding cell-free blanks. Each condition was assessed in five technical replicate wells.

### 2.12 RT-qPCR

After reperfusion, intestinal tissues were rapidly collected from the same anatomical site in each mouse, snap-frozen in liquid nitrogen, and stored at −80 °C. Total RNA was extracted from intestinal tissue using a total RNA extraction reagent (Leagene, China, NR0002), and the RNA concentration was measured using a microvolume nucleic acid analyzer. Equal amounts of RNA were processed using the HiScript II Q RT SuperMix for qPCR (+gDNA wiper) reverse transcription kit (Vazyme, China, R223-01) to remove residual genomic DNA and synthesize cDNA. The cDNA was diluted 10-fold and used as the qPCR template.

qPCR was performed using ChamQ Universal SYBR qPCR Master Mix (Vazyme, China, Q711-02). The amplification program was as follows: initial denaturation at 95 °C for 30 s; 40 cycles of 95 °C for 10 s and 60 °C for 30 s. After amplification, melting curve analysis was performed to verify product specificity (Bustin et al., 2025). Primer sequences are provided in Supplementary Material 5 (Sangon, China). β-Actin was used as the reference gene, and three biological replicates were performed for each sample (Bustin et al., 2025). The corresponding mRNA expression levels were calculated using the 2^−ΔΔCt method and expressed as fold change relative to sham + vehicle (Bustin et al., 2025).

### 2.13 Western blotting

Fresh mouse intestinal tissues were washed with precooled PBS and blotted dry. Equal amounts of tissue were weighed and added to RIPA lysis buffer containing a protease inhibitor mixture (Solarbio, China, R0010), followed by homogenization and lysis on ice for 30 min at a ratio of 1:10 (mg:μL). After centrifugation at 12,000 × g for 15 min at 4 °C, the supernatant was collected, and the protein concentration was measured using a BCA protein assay kit (Beyotime, China, P0010). Samples were mixed with 5× SDS loading buffer and denatured in a metal bath at 95 °C for 5 min. Equal amounts of protein (20–40 μg/well) were separated by 8%–12% SDS-polyacrylamide gel electrophoresis (SDS-PAGE), and the proteins were transferred to a polyvinylidene fluoride (PVDF) membrane (Millipore) using a semi-dry transfer method.

The membranes were blocked in TBST containing 5% nonfat dry milk (Solarbio, China, A8020) for 1–2 h at room temperature. The corresponding primary antibodies were then added: ABCG2 (Proteintech, China, 27286-1-AP, 1:1,000); CHOP (Proteintech, China, 15204-1-AP, 1:1,000); Occludin (Proteintech, China, 27260-1-AP, 1:1,000); Claudin-1 (Proteintech, China, 13050-1-AP, 1:1,000); ZO-1 (Proteintech, China, 21773-1-AP, 1:1,000) and NRF2 (Proteintech, China, 33123-1-AP, 1:1,000). All primary antibodies were rabbit IgG antibodies, and the membranes were incubated overnight at 4 °C. The next day, the membranes were washed 3 times with TBST for 10 min each and then incubated with HRP-Goat Anti-Rabbit Recombinant Secondary Antibody (H + L) (Proteintech, China, RGAR001, 1:10,000) at 37 °C for 2 h, followed by another TBST wash. Protein signals were detected using an enhanced chemiluminescence (ECL) reagent, and images were acquired using a chemiluminescence imaging system. Grayscale values were quantified using ImageJ and normalized to *β*-actin as the reference protein (Omondi et al., 2024). All Western blotting experiments included three biological replicates (*n* = 3).

### 2.14 H&E staining

After reperfusion, ileal tissues of equal length were collected from the same anatomical site in mice from all groups. The tissues were rinsed with precooled PBS, trimmed, and fixed in 4% tissue and cell fixative (Solarbio, P110) for 24 h at room temperature. All tissues were dehydrated through a graded series, cleared in xylene, and embedded in paraffin. A consistent embedding orientation was used to ensure sample comparability. Serial 4 μm paraffin sections were prepared and baked at 60 °C for 2 h. Adjacent sections were used for H&E staining and IHC.

For H&E staining, paraffin sections were first deparaffinized in xylene I and II for 10 min each. The sections were then rehydrated sequentially in absolute ethanol I and II and 95%, 85%, and 75% ethanol for 5 min each, followed by thorough washing with distilled water. A hematoxylin and eosin staining kit (Beyotime, China, C0105) was used. The sections were stained with hematoxylin for 5 min, differentiated with hydrochloric acid-ethanol, blued, and then stained with eosin for 2 min. After staining, the sections were dehydrated through a graded series, cleared, mounted with neutral resin, and examined under a light microscope for changes in intestinal mucosal structure.

Images were acquired using uniform microscope settings, and pathological changes, including villus integrity, epithelial shedding, crypt injury, mucosal hemorrhage, and inflammatory infiltration, were examined. The degree of intestinal mucosal injury was semiquantitatively evaluated using the classic Chiu’s histological scoring system (Wang et al., 2024a). Based on villus structural integrity, the degree of epithelial cell shedding, the extent of mucosal injury, and damage to the lamina propria, this scoring system classifies intestinal tissue injury into grades 0–5 (Wang et al., 2023b). The criteria were as follows: grade 0, intact villus structure, normal mucosal morphology, and no obvious pathological changes; grade 1, formation of a subepithelial Gruenhagen space at the villus tip, usually accompanied by capillary congestion; grade 2, further expansion of the subepithelial space, with moderate separation between the epithelial layer and lamina propria; grade 3, obvious epithelial lifting along both sides of the villi, with exposure or shedding at the tips of some villi; grade 4, extensive shedding of the villus epithelium, exposure of the lamina propria, capillary dilation, and increased cellular infiltration into the lamina propria; and grade 5, digestion and disintegration of the lamina propria, with hemorrhage and ulceration (Li et al., 2022). Five experimental animals were randomly selected from each group, and one section from each animal was stained.

### 2.15 Immunohistochemistry

After deparaffinization and graded rehydration as described above, the paraffin sections were placed in 10 mM sodium citrate antigen retrieval solution at pH 6.0 (Solarbio, C1032) and heated in a microwave for antigen retrieval for 15 min. After cooling to room temperature, the sections were washed 3 times with PBS for 5 min each. Endogenous peroxidase activity was blocked by incubation with 3% hydrogen peroxide in methanol for 10 min at room temperature in the dark. After rinsing with PBS, the sections were blocked with 5% goat serum blocking solution (Beyotime, C0265) for 30 min at room temperature to reduce nonspecific binding.

The sections were incubated with a rabbit anti-ABCG2 primary antibody (Abiowell, AWA10989, 1:200) overnight at 4 °C in a humidified chamber. After thorough washing with PBS, an HRP-labeled secondary antibody (Beyotime, A0208, 1:500) was added, and the sections were incubated at 37 °C for 1 h. Color was developed using a DAB chromogenic kit (Beyotime, P0202), and the reaction was monitored under a microscope and stopped at an appropriate time. Nuclei were counterstained with hematoxylin. After differentiation, bluing, graded dehydration, and clearing, the sections were mounted with neutral resin.

Images were acquired under uniform imaging conditions. All IHC sections were imaged using identical microscope acquisition settings throughout the experiment. Five animals were randomly selected from each experimental group, and one stained section from each animal was included for quantitative analysis (Schmied et al., 2024). Multiple non-overlapping fields were acquired from each section using the same magnification, illumination, exposure, and image-acquisition parameters. For quantitative analysis, hematoxylin and DAB signals were separated using the Color Deconvolution function in ImageJ, and all subsequent measurements were performed exclusively on the DAB channel. Before batch analysis, the DAB optical-density threshold was calibrated using representative negative, weakly positive, and strongly positive samples. A fixed DAB optical-density threshold of 0.20 was then applied uniformly to all images and was not adjusted for individual sections or experimental groups. Regions containing obvious tissue folds, tears, debris, or other staining artifacts were excluded from the analysis. ABCG2 immunoreactivity was quantified using two complementary parameters: the percentage of DAB-positive tissue area (% positive area) and the mean optical density (MOD) of the DAB-positive signal. MOD values were derived from the grayscale intensity of the separated DAB channel after conversion to optical-density values. Measurements from multiple non-overlapping fields within each animal were averaged to generate a single biological value for that animal, and these animal-level values were subsequently used for between-group statistical comparisons. To minimize observer bias, a blinded analysis procedure was implemented. Before image acquisition and quantitative analysis, all sections were randomly coded by an investigator who was not involved in image collection or ImageJ analysis, and all information identifying the experimental groups was concealed. Investigators responsible for microscopic image acquisition and ImageJ-based quantification remained unaware of group allocation throughout the entire analytical process. After all measurements had been completed and the quantitative dataset had been finalized and locked, group identities were decoded by a third investigator before statistical analysis.

### 2.16 Immunofluorescence staining

IEC-6 cells were seeded on sterile coverslips and subjected to the indicated treatments. After treatment, the cells were washed 3 times with PBS and fixed with 4% paraformaldehyde for 15 min at room temperature. The cells were then washed with PBS and permeabilized with 0.2% Triton X-100 for 10 min. After three additional washes with PBS, nonspecific binding was blocked with 5% goat serum for 30 min at room temperature. The cells were subsequently incubated with a rabbit anti-NRF2 primary antibody (Proteintech, China, 33123-1-AP, 1:200) overnight at 4 °C in a humidified chamber.

The following day, the cells were washed three times with PBS for 5 min each and incubated with an appropriate fluorophore-conjugated goat anti-rabbit IgG secondary antibody (1:500) for 1 h at room temperature in the dark. After washing, nuclei were counterstained with DAPI for 5 min, followed by three washes with PBS. Coverslips were mounted using an antifade mounting medium and examined using a fluorescence microscope. All images were acquired using identical microscope settings throughout the experiment, including the same magnification, illumination intensity, exposure time, gain, and image-acquisition parameters.

For quantitative analysis of NRF2 subcellular localization, multiple non-overlapping fields were acquired from each biological replicate under identical imaging conditions. Nuclear and cytoplasmic NRF2 fluorescence intensities were quantified using ImageJ, and the nuclear-to-cytoplasmic fluorescence intensity ratio was calculated for each cell. Values from multiple cells within the same biological replicate were averaged and used as a single biological value for subsequent statistical analysis.

To minimize observer bias, a blinded analysis procedure was implemented. Before image acquisition and quantitative analysis, all samples were randomly coded by an investigator who was not involved in fluorescence imaging or ImageJ analysis, and all information identifying the experimental groups was concealed. Investigators responsible for image acquisition and quantitative analysis remained unaware of group allocation throughout the analytical process. After all measurements had been completed and the quantitative dataset had been finalized and locked, group identities were decoded by a third investigator before statistical analysis.

### 2.17 Statistical analysis

Three mice per group were used for RT-qPCR and Western blotting (*n* = 3), whereas five mice per group were used for H&E staining, IHC (*n* = 5) and IF (*n* = 8). Data collection and analysis were performed in a single-blind manner. Data are presented as the mean ± standard deviation. Comparisons among groups were performed using one-way analysis of variance, followed by Tukey’s *post hoc* multiple-comparison test. *P* < 0.05 was considered statistically significant.

## 3 Results

### 3.1 Screening and functional enrichment analysis of core candidate genes for corilagin against IIRI via regulating ERS

To examine potential molecular mechanisms associated with the effects of Corilagin on IIRI and ERS, this study integrated 1,469 predicted target genes of Corilagin, 6514 ERS-related genes, and 7445 IIRI-related genes. Among them, 737 genes were unique to Corilagin, 2,641 were unique to ERS, and 3,537 were unique to IIRI; 472 genes were shared by Corilagin, ERS, and IIRI and were used as the core candidate gene set for subsequent analyses (Figure 2A). GO functional enrichment analysis showed that these genes were enriched in biological processes such as GO:0050727 (regulation of inflammatory response), GO:0042060 (wound healing), and GO:0071375 (cellular response to peptide hormone stimulus) (Figure 2B). These enrichments link the candidate gene set to inflammatory regulation, wound healing, and related responses after IIRI.

**FIGURE 2:**
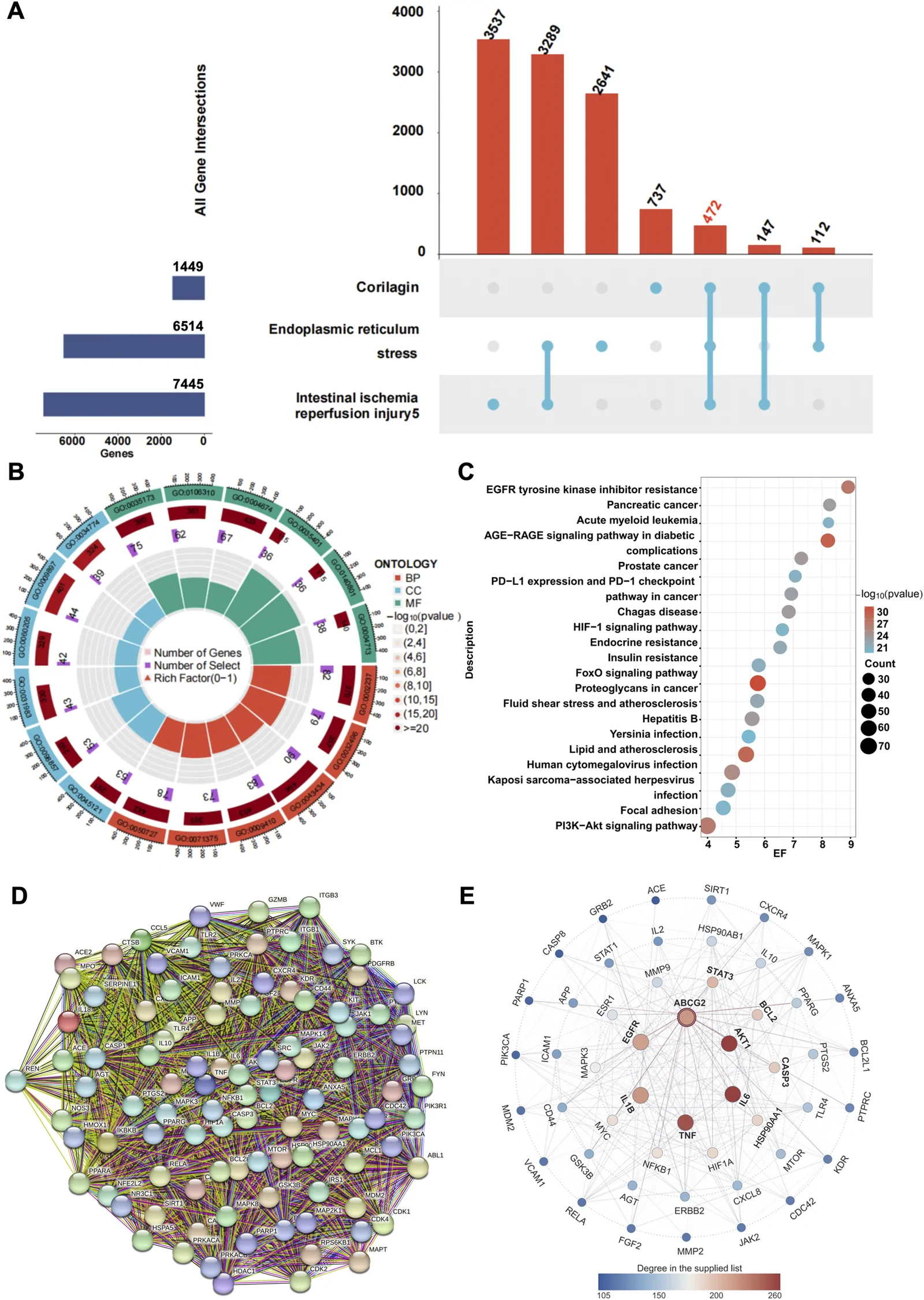
Potential protective mechanisms of Corilagin through regulation of ERS. **(A)** Identification of overlapping targets among Corilagin-related genes, ERS related genes, and IIRI associated genes using Venn diagram and UpSet plot analysis. **(B)** Gene Ontology (GO) enrichment analysis of the intersecting target genes. **(C)** Kyoto Encyclopedia of Genes and Genomes (KEGG) pathway enrichment analysis of candidate targets. **(D)** Protein–protein interaction (PPI) network analysis of the candidate target genes. **(E)** Network visualization of these target genes identified from the PPI analysis.

KEGG pathway enrichment analysis further showed these genes were significantly enriched in several stress and disease-related signaling pathways, including the PI3K–Akt signaling pathway, AGE–RAGE signaling pathway in diabetic complications, and HIF-1ɑ signaling pathway (Figure 2C). A protein–protein interaction (PPI) network was constructed based on the intersecting genes, and the results showed dense interactions among the proteins (Figure 2D). Further topological analysis placed ABCG2, AKT1, IL6, TNF, IL1B and EGFR at central positions in the PPI network. These proteins were therefore considered candidate central nodes associated with ERS and inflammation related processes; their functional roles require further validation (Figure 2E).

### 3.2 Machine learning-based multidimensional screening of core genes regulated by corilagin in ERS and IIRI

Subsequently, a machine learning-based strategy was used to further screen the candidate target genes set and identify core genes involved in the regulation of ERS by Corilagin to ameliorate IIRI. Two transcriptomic datasets were selected from the GEO database: the human dataset GSE37013 and the mouse dataset GSE96733. A total of six comparison groups were defined according to different time points and the pooled time windows within the datasets: reperfusion 0.5 h vs. Normal, reperfusion 2 h vs. Normal, and pooled reperfusion 0.5 + 2 h vs. Normal in the human dataset; and reperfusion 3 h vs. WT, reperfusion 6 h vs. WT, and pooled reperfusion 3 + 6 h vs. WT in the mouse dataset; these groups were used to capture the dynamic gene expression characteristics during IIRI.

Differential expression analysis was performed separately for the six comparison groups, yielding 7, 11, 9, 792, 415, and 481 downregulated differentially expressed genes (DEGs), respectively, and 39, 52, 45, 818, 556, and 594 upregulated DEGs, respectively (Figure 3A). Differentially expressed genes from each time window in the human and mouse datasets were combined and deduplicated, yielding 1,126 genes; intersection of these genes with the 472 genes described above ultimately yielded 73 overlapping genes that responded to IIRI and were also associated with the predicted targets of Corilagin and ERS (Figure 3B). The heatmap showed significant differences in the expression profiles of these genes between IIRI and normal samples (Figure 3C).

**FIGURE 3:**
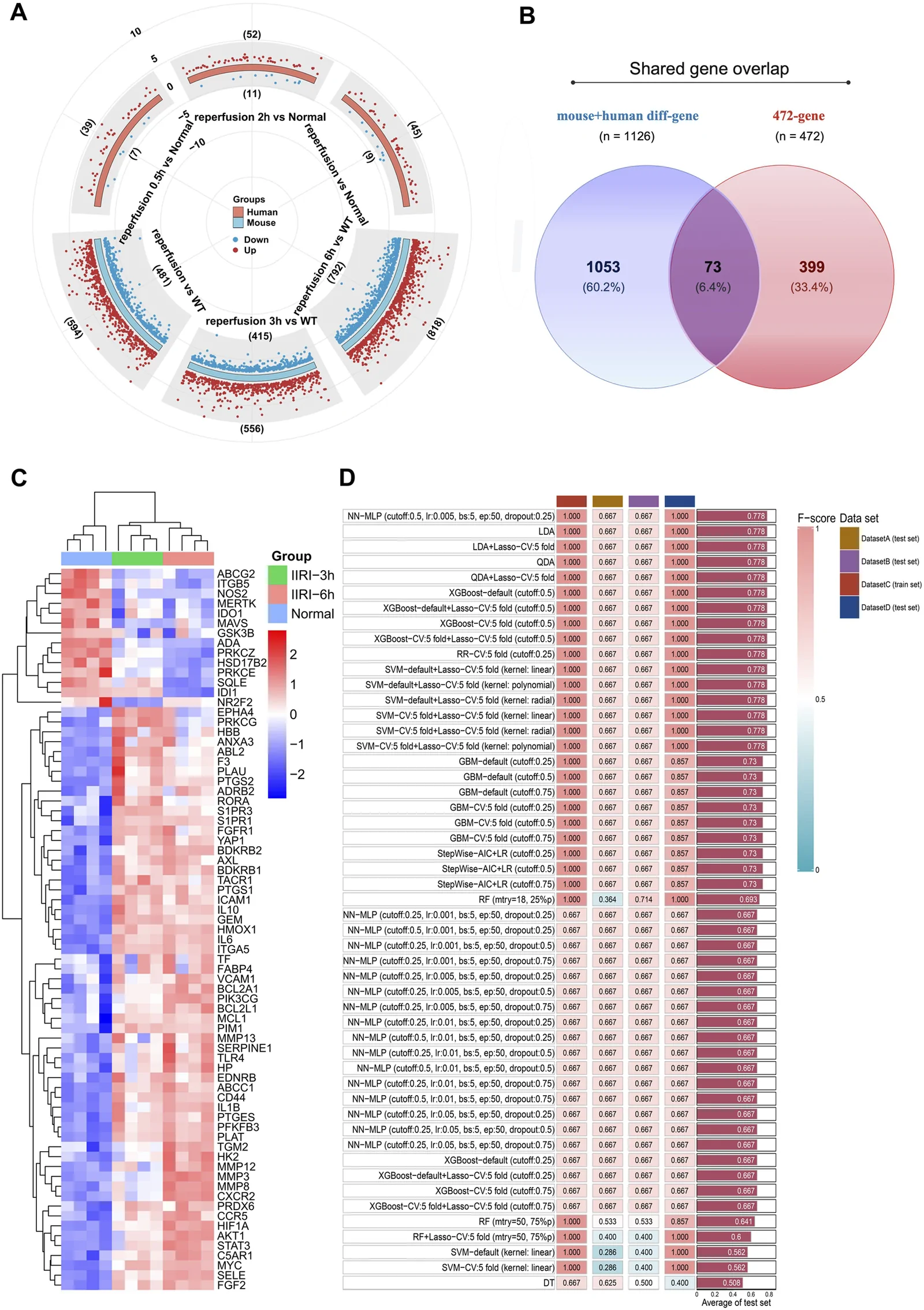
Identification of key molecular signatures associated with IIRI through integrated transcriptomic analysis and machine learning approaches. **(A)** Circular visualization of differentially expressed genes identified from human and mouse IIRI datasets. **(B)** Venn diagram showing the overlapping differentially expressed genes between human and mouse IIRI datasets. **(C)** Heatmap visualization of the expression profiles of the 73 genes across different experimental groups. **(D)** Machine learning-based screening of diagnostic core genes using multiple algorithms. Model performance was evaluated based on different classification approaches and datasets.

To more accurately identify core genes from the 73 overlapping gene set and explore potential targets associated with Corilagin-mediated regulation of ERS and improvement of IIRI, machine learning algorithms were used for multidimensional screening and validation. Based on the datasets described above, 15 types of machine learning classifiers were trained at multiple probability thresholds, yielding 207 optimized models. Because of the limited availability of datasets, two analytical modes comprising four machine learning strategies, S1–S4, were used: in S1, all samples from the human dataset were used as the training set and all samples from the mouse dataset were used as the validation set; in S2, all samples from the mouse dataset were used as the training set and all samples from the human dataset were used as the validation set; in S3, the human data were grouped by IIRI time, with Normal serving as the common control group, and the mouse data were also grouped by IIRI time, with WT serving as the common control group. Four sub-data matrices were ultimately obtained, including two human and 2 mouse matrices. The human IIRI time-point data were used as the training set, and the other three matrices were used as validation sets; in S4, under the IIRI time grouping used in S3, the mouse IIRI time-point data were used as the training set and the other matrices were used as validation sets (Supplementary Material 6); all four strategies used the F-score as the evaluation criterion, and models with scores greater than 0.5 were selected for computational validation (Figure 3D).

### 3.3 Screening and expression pattern analysis of key core genes in IIRI

Gene importance was ranked using four machine learning strategies, and the top 35 genes from each strategy were extracted and visualized in bar plots (Figure 4A). These rankings were integrated with the preceding intersection analysis, and the machine learning results were cross-referenced with the previous screening results, ultimately identifying 12 overlapping core genes and the adjusted P values of all 12 core genes were <0.05 (Supplementary Material 7).

**FIGURE 4:**
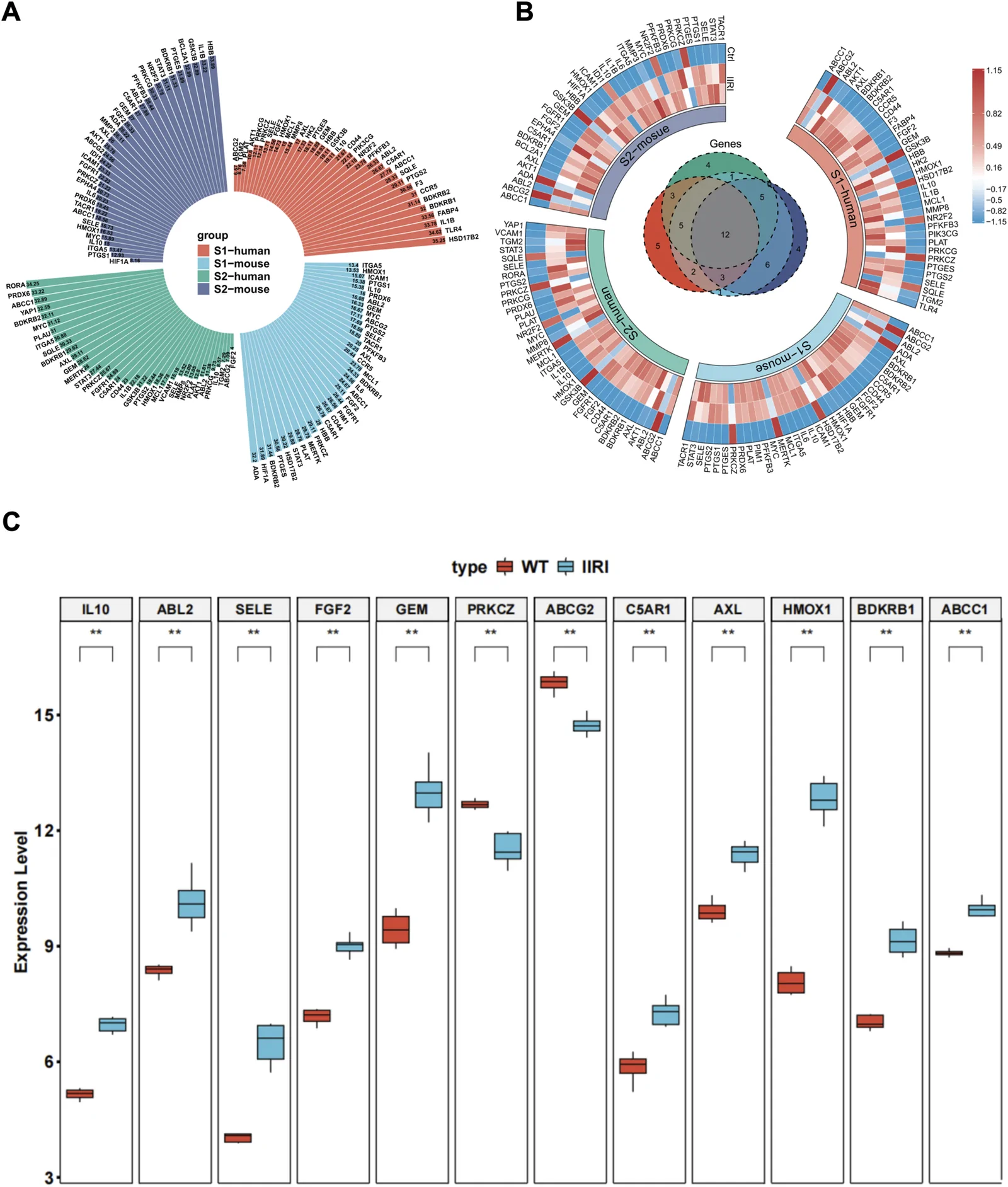
Identification and validation of key genes associated with IIRI. **(A)** Circular visualization of differentially expressed genes identified from integrated human and mouse IIRI datasets. **(B)** Comparative analysis of core genes identified from different datasets using Venn diagram and circular heatmap visualization. **(C)** Expression validation of core genes. **P < 0.01.

A circular heatmap was used to visualize the differential expression patterns of the 12 core genes in the IIRI dataset (Figure 4B). Between-group differential expression analysis was then used to compare their expression levels between the IIRI and control groups. Compared with the control group, IL10, ABL2, SELE, FGF2, GEM, C5AR1, AXL, HMOX1, BDKRB1, and ABCC1 were significantly upregulated in the IIRI group (all *P* < 0.01), whereas ABCG2 and PRKCZ were significantly downregulated (both *P* < 0.01; Figure 4C). These results show that IIRI was associated with altered expression of inflammatory mediators, signaling molecules, and transporters.

### 3.4 Functional enrichment and network analysis of core genes in IIRI

To examine the biological functions associated with the 12 core genes in IIRI, GO functional enrichment and KEGG pathway enrichment analyses were performed. GO functional enrichment analysis showed that these genes were enriched in terms such as leukocyte migration, membrane microdomain, and basolateral plasma membrane (Figure 5A). These enrichments link the core genes to processes related to immune cell recruitment, epithelial polarity, and homeostasis.

**FIGURE 5:**
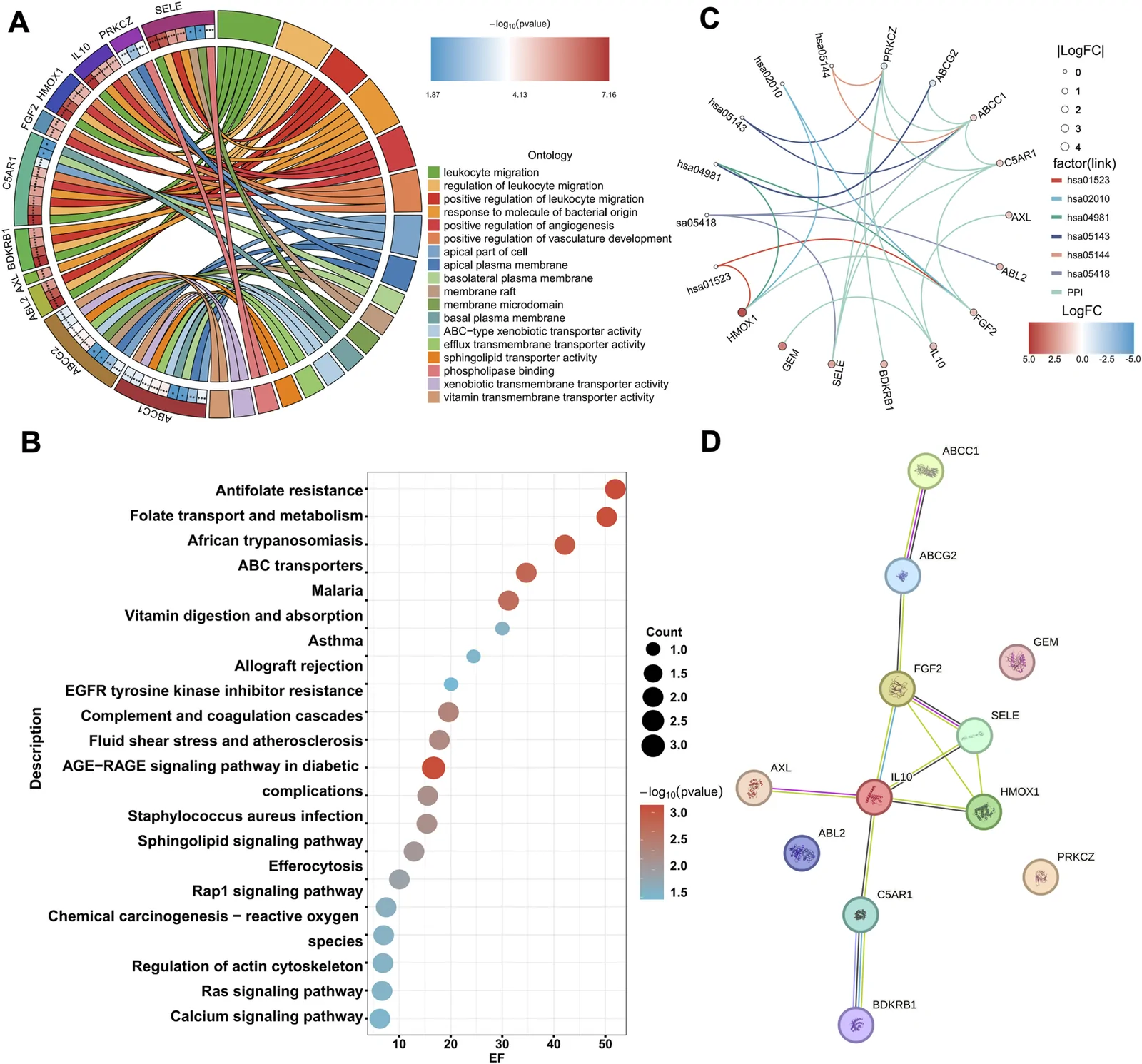
Functional enrichment and network analysis of core genes associated with IIRI. **(A)** Gene Ontology (GO) enrichment analysis of core genes visualized by chord diagram. **(B)** Kyoto Encyclopedia of Genes and Genomes (KEGG) pathway enrichment analysis of core genes. **(C)** Regulatory network analysis of core genes and associated microRNAs. **(D)** PPI network analysis of core genes.

KEGG pathway enrichment analysis showed that the 12 core genes were significantly enriched in pathways including ABC transporters and EGFR tyrosine kinase inhibitor resistance (Figures 5B,C). PPI network analysis of the encoded proteins showed possible functional relationships among these proteins (Figure 5D). In summary, the 12 genes represent candidate points of convergence associated with ERS, inflammatory responses, and barrier-related processes in IIRI.

### 3.5 Corilagin partially reverses the abnormal expression of 12 core genes in IIRI

To experimentally evaluate the bioinformatics results and the effects of Corilagin on the expression of these core genes, RT-qPCR was performed in an animal model of IIRI. The expression patterns observed by RT-qPCR were consistent with the bioinformatics predictions. Compared with sham + vehicle, 10 genes, including BDKRB1, ABL2, AXL, ABCC1, C5AR1, FGF2, GEM, HMOX1, IL10, and SELE, were significantly upregulated in IIRI + vehicle, with significance levels ranging from *P* < 0.05 to *P* < 0.001. These changes were consistent with altered transcription of genes related to inflammatory responses and cellular stress during IIRI. In contrast, ABCG2 and PRKCZ expression was significantly downregulated in IIRI + vehicle compared with sham + vehicle (*P* < 0.05 and *P* < 0.001, respectively), showing lower expression of these selected protective and homeostasis-related genes.

Notably, gene-expression dysregulation under these pathological conditions was partially alleviated in IIRI + Corilagin after pretreatment with 30 mg/kg Corilagin. Compared with IIRI + vehicle, IIRI + Corilagin had significantly lower transcript levels of the 10 upregulated genes described above, with significance levels ranging from *P* < 0.05 to *P* < 0.001. The expression of most genes approached the levels in sham + vehicle, an expression pattern consistent with reduced inflammation and partial recovery of tissue homeostasis (Chu et al., 2023; Zhang et al., 2023c). IIRI + Corilagin also had significantly higher expression of ABCG2 (*P* < 0.05) and PRKCZ (*P* < 0.05) than IIRI + vehicle, consistent with partial restoration of their expression (Figure 6).

**FIGURE 6:**
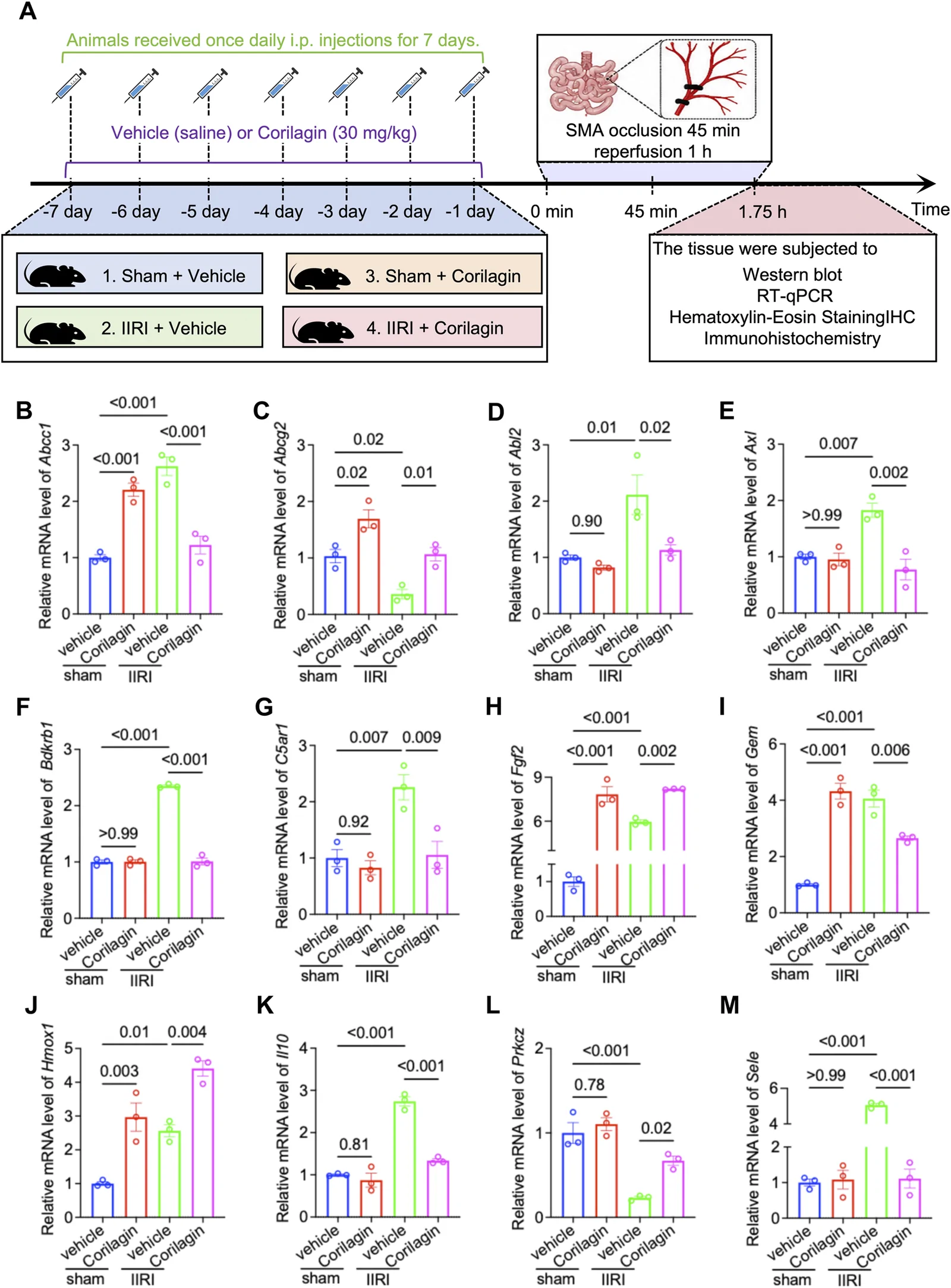
*In vivo* evaluation of the expression of the screened core genes. **(A)** Schematic illustration of the experimental design for Corilagin pretreatment in a mouse IIRI (IIRI) model. Mice received daily intraperitoneal administration of vehicle or Corilagin (30 mg/kg) for seven consecutive days, followed by superior mesenteric artery (SMA) occlusion for 45 min and reperfusion for 1 h. Intestinal tissues were collected for subsequent molecular and histological analyses (B–M) Relative mRNA expression levels of IIRI associated hub genes in intestinal tissues determined by RT-qPCR, including *Abcc1* **(B)**, *Abcg2* **(C)**, *Abl2* **(D)**, *Axl* **(E)**, *Bdkrb1* **(F)**, *C5ar1* **(G)**, *Fgf2* **(H)**, *Gem* **(I)**, *Hmox1* **(J)**, *Il10* **(K)**, *Prkcz* **(L)**, and *Sele* **(M)** among different experimental groups.

### 3.6 Protective effects of corilagin on the intestinal mucosa in IIRI and regulation of ABCG2 expression

To further evaluate the intestinal-protective effects of Corilagin and examine the selected targets at the tissue and protein levels, histopathological staining and protein quantification were performed. H&E staining showed severe intestinal histopathological damage in IIRI + vehicle, mainly characterized by disruption of the villus structure, extensive epithelial shedding, disorganization of the basal gland structure, and loss of tissue integrity. Compared with IIRI + vehicle, histopathological damage was significantly reduced in IIRI + Corilagin, with better preservation of intestinal villi and more regular basal gland arrangement (Figures 7A,B). These findings support preservation of intestinal mucosal and barrier morphology by Corilagin pretreatment in IIRI.

**FIGURE 7:**
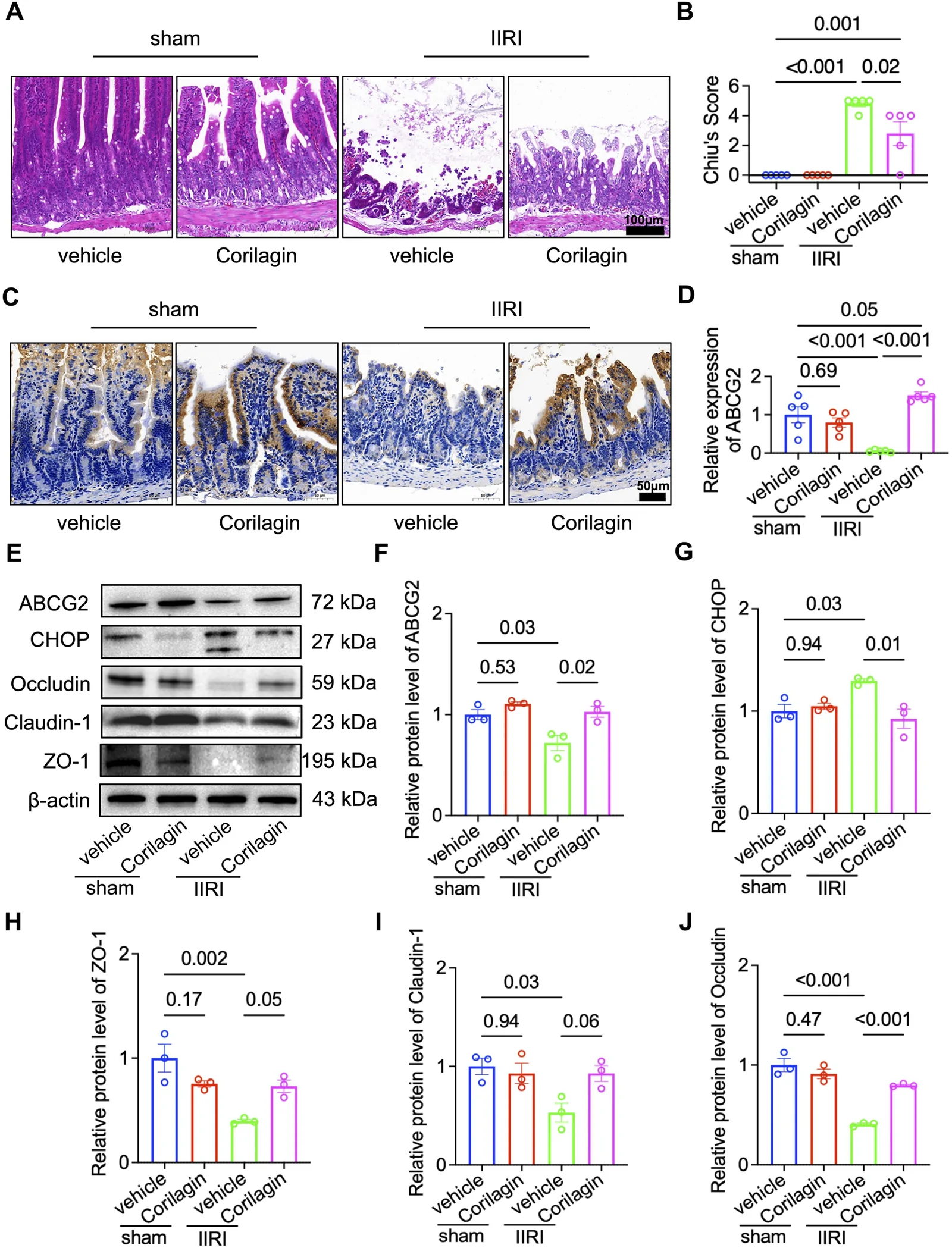
Corilagin alleviates IIRI by improving intestinal mucosal integrity and restoring epithelial barrier-associated proteins. **(A)** Representative hematoxylin and eosin (H and E) staining images of intestinal tissues from sham and IIRI mice treated with vehicle or Corilagin. **(B)** Quantification of intestinal mucosal injury according to the Chiu’s scoring system. **(C)** Representative immunohistochemical staining images showing the expression of ABCG2 in intestinal tissues among different experimental groups. **(D)** Quantitative analysis of ABCG2 expression based on immunohistochemical staining. **(E)** Western blot analysis of ABCG2, CHOP, and tight junction-associated proteins (Occludin, Claudin-1, and ZO-1) in intestinal tissues **(F–J)** Densitometric quantification of protein expression levels of ABCG2 **(F)**, CHOP **(G)**, ZO-1 **(H)**, Claudin-1 **(I)**, and Occludin **(J)**, normalized to β-actin.

Based on reports that ABCG2 is a key transporter associated with intestinal protection, this study further examined changes in its protein expression by immunohistochemistry (IHC) and Western blotting. The IHC results showed that ABCG2 was mainly expressed at the apical brush border of intestinal epithelial cells in sham + vehicle. Compared with sham + vehicle, ABCG2 staining intensity was significantly lower in the intestinal tissue of IIRI + vehicle (*P* < 0.01), indicating lower ABCG2 abundance after IIRI. ABCG2 protein expression was significantly higher in IIRI + Corilagin than in IIRI + vehicle (*P* < 0.01; Figures 7C,D).

Western blot quantification was consistent with the IHC results. Compared with sham + vehicle, ABCG2 protein levels were significantly lower in IIRI + vehicle (*P* < 0.05); compared with IIRI + vehicle, ABCG2 protein levels were significantly higher in IIRI + Corilagin (*P* < 0.001; Figures 7E,F). In addition, Occludin, Claudin-1, and ZO-1 protein levels were lower in IIRI + vehicle, consistent with disruption of intestinal tight junctions in IIRI.Quantitative analyses of CHOP, ZO-1, Claudin-1, and Occludin are shown in Figures 7G-J. Compared with IIRI + vehicle, the expression of all three tight junction proteins was significantly higher in IIRI + Corilagin (*P* < 0.001; Figures 7E,F). These findings support an association between Corilagin pretreatment and preservation of barrier-related protein expression.

In summary, IIRI + Corilagin showed higher ABCG2 and tight junction protein expression and better-preserved intestinal epithelial morphology than IIRI + vehicle. These findings support an association between Corilagin pretreatment and protection against IIRI.

### 3.7 Molecular simulations support a predicted stable binding mode between corilagin and NRF2

NRF2 is a transcriptional regulator of cellular anti-stress responses, and studies in non-IIRI systems have linked NRF2 signaling to the expression of ABC transporters, including ABCG2. This study therefore examined the predicted interaction between Corilagin and NRF2 as a possible mechanism associated with higher ABCG2 expression. Molecular docking predicted a preferred pose of Corilagin on the modeled NRF2 chain from AlphaFold DB model ID: AF-Q16236-F1, with a predicted binding energy of −8.7 kcal/mol (Figure 8A). The predicted pose included multiple hydrogen-bond and hydrophobic contacts at the modeled docking site. Static molecular docking provides only a predicted pose and cannot confirm direct binding or transcriptional activation *in vivo*; dynamic simulations and functional experiments are still required.

**FIGURE 8:**
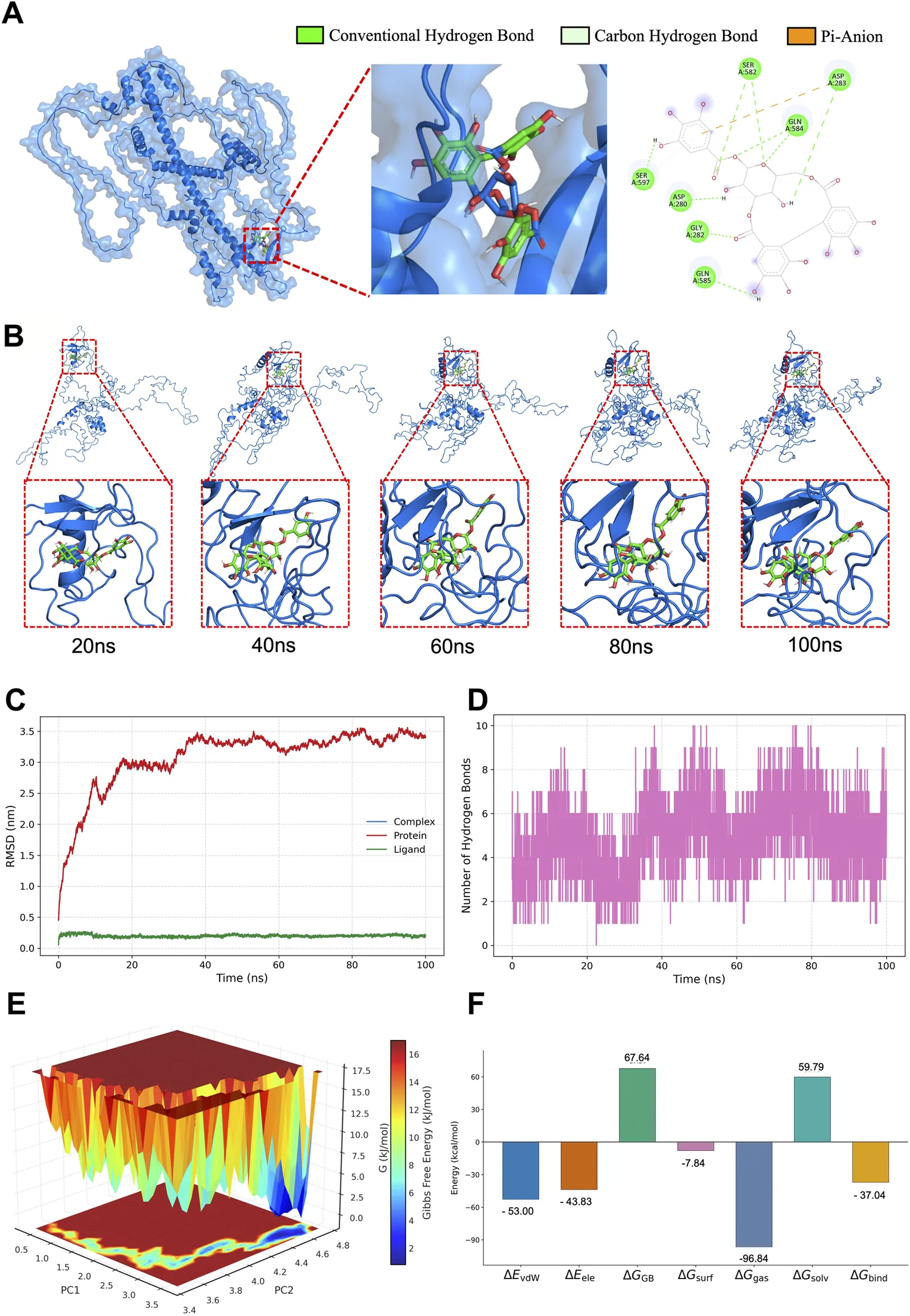
Molecular docking and molecular dynamics simulation analysis of Corilagin interacting with the target protein. **(A)** Molecular docking analysis showing the predicted binding mode and interaction pattern between Corilagin and the target protein. The binding affinity and key intermolecular interactions are indicated. **(B)** Representative snapshots of the Corilagin–target protein complex during a 100 ns molecular dynamics (MD) simulation. **(C)** Root-mean-square deviation (RMSD) profiles of the protein, protein–ligand complex, and ligand during the 100-ns MD simulation. **(D)** Hydrogen bond analysis of the Corilagin–target protein complex throughout the MD simulation. **(E)** Gibbs free energy landscape analysis of the protein–ligand complex based on principal component analysis. **(F)** MM/GBSA energy decomposition calculated from snapshots extracted from the equilibrated 60–100 ns trajectory segment. ΔGMM/GBSA represents the end-state energy estimate before entropy correction.

To further characterize the dynamic stability of the predicted Corilagin–NRF2 complex, a 100 ns molecular dynamics (MD) simulation was performed. During the 100-ns MD simulation, the global RMSD of the protein and the protein–ligand complex increased substantially relative to the starting structure and subsequently fluctuated around a later plateau. The protein and complex RMSD profiles were nearly superimposable, indicating that the large global RMSD predominantly reflected conformational rearrangement of NRF2 rather than displacement attributable to the ligand. In contrast, ligand-centered analysis showed substantially smaller fluctuations. After alignment of the trajectory to the protein or binding-site backbone, Corilagin remained associated with the initially predicted binding region throughout the sampled trajectory. Consistently, PBC-corrected representative structures at 20, 40, 60, 80, and 100 ns showed no complete dissociation of Corilagin from the predicted binding region during the 100-ns simulation (Figures 8B,C). Hydrogen-bond analysis showed persistent interactions between Corilagin and NRF2 during the simulation; 1–4 hydrogen bonds were present in most frames, supporting persistent local interactions between Corilagin and NRF2 (Figure 8D).

Free-energy landscape analysis revealed a dominant low-energy conformational basin, suggesting that this predicted pose occupied a low-energy region with limited conformational deviation in the simulation (Figure 8E). To reduce the influence of the initial conformational relaxation, MM/GBSA analysis was restricted to the equilibrated 60–100 ns segment of the trajectory. A total of 401 snapshots were analyzed. The van der Waals and electrostatic contributions were −53.00 and −43.83 kcal/mol, respectively, whereas the polar-solvation and nonpolar-solvation contributions were 67.64 and −7.84 kcal/mol, respectively. These contributions yielded an MM/GBSA energy estimate before entropy correction of −37.04 kcal/mol, indicating a favorable predicted energetic interaction over the equilibrated trajectory segment (Figure 8F). Interaction entropy analysis yielded an estimated -TASIE of 21.71 kcal/mol. However, the standard deviation of the interaction energy was 11.77 kcal/mol, substantially exceeding the recommended reliability threshold of approximately 3.6 kcal/mol. Accordingly, although the IE-corrected estimate was −15.34 kcal/mol, this value was not interpreted quantitatively, and the uncorrected stable-window MM/GBSA estimate was retained as the primary end-state energetic metric.

### 3.8 NRF2 and ABCG2 contribute to corilagin-mediated preservation of tight junction proteins in IEC-6 cells

To further examine whether the predicted Corilagin–NRF2 interaction identified by molecular docking and molecular dynamics simulations could be supported at the cellular level, a cellular thermal shift assay (CETSA) was performed in IEC-6 cells. Corilagin treatment increased the thermal stability of NRF2, providing cellular target-engagement evidence consistent with an interaction between Corilagin and NRF2 (Supplementary Material 8). To extend the *in vivo* findings and examine the involvement of NRF2 and ABCG2 in the effects of Corilagin, experiments were performed in IEC-6 cells using siRNA-mediated knockdown and an OGD/R model (Figure 9A). CCK-8 analysis showed that cell viability remained close to the vehicle-control level after 24 h of treatment with 0.25–0.75 μM Corilagin, whereas viability was significantly reduced at 1.0–1.5 μM (all *P* < 0.001 vs. vehicle; Figure 9B). Accordingly, 0.75 μM Corilagin was selected for subsequent experiments.

**FIGURE 9:**
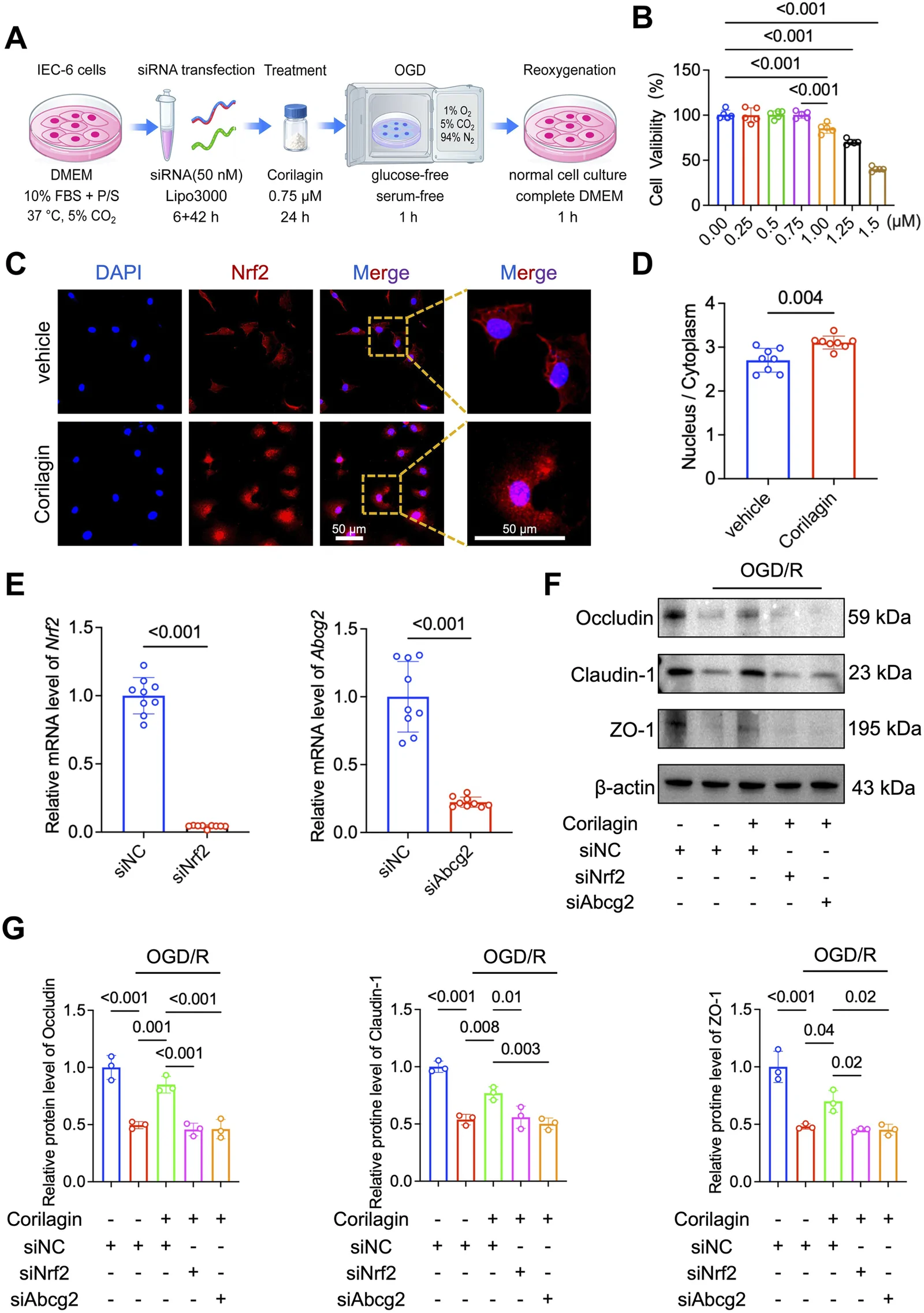
Effects of Corilagin on NRF2 nuclear accumulation and tight junction protein expression in IEC-6 cells. **(A)** Schematic illustration of the experimental workflow, including siRNA transfection (50 nM, 48 h), Corilagin pretreatment (0.75 μM, 24 h), oxygen–glucose deprivation for 1 h, and reoxygenation for 1 h. **(B)** Relative viability of IEC-6 cells treated with the indicated concentrations of Corilagin for 24 h under basal culture conditions, assessed using the CCK-8 assay and expressed relative to the vehicle control. **(C)** Representative immunofluorescence images showing NRF2 localization in vehicle- and Corilagin-treated IEC-6 cells. NRF2 is shown in red, and nuclei are counterstained with DAPI in blue. Boxed regions are enlarged in the rightmost panels. Scale bars, 50 μm. **(D)** Quantification of the nuclear-to-cytoplasmic NRF2 fluorescence ratio. **(E)** Relative mRNA levels of Nrf2 and *Abcg2* following transfection with the corresponding siRNAs, determined by RT-qPCR and expressed relative to siNC. **(F)** Representative Western blots of Occludin, Claudin-1, and ZO-1 in the siNC, siNC + OGD/R, siNC + OGD/R + Corilagin, siNrf2 + OGD/R + Corilagin, and siAbcg2 + OGD/R + Corilagin groups. *β*-Actin served as the loading control. **(G)** Densitometric quantification of the proteins shown in **(F)**, normalized to *β*-actin and expressed relative to the siNC group. Data are presented as the mean ± SD. The CCK-8 assay included five technical replicate wells per condition, and Western blotting included three biological replicates. *P* values for the indicated comparisons are shown.

Immunofluorescence analysis showed increased nuclear accumulation of NRF2 following Corilagin treatment, with a significantly higher nuclear-to-cytoplasmic fluorescence ratio than in vehicle-treated cells (*P* = 0.004; Figures 9C,D). To further examine the contributions of NRF2 and ABCG2, IEC-6 cells were transfected with siNrf2 or siAbcg2. RT-qPCR confirmed that the corresponding *Nrf2* and *Abcg2* mRNA levels were markedly reduced compared with those in the siNC group (both *P* < 0.001; Figure 9E).

Western blotting showed that OGD/R significantly decreased Occludin, Claudin-1, and ZO-1 protein levels compared with the siNC group (all *P* < 0.001). Corilagin pretreatment partially restored the expression of these proteins compared with siNC + OGD/R (*P* = 0.001, *P* = 0.008, and *P* = 0.04, respectively). However, the levels of all three proteins were significantly lower in both the siNrf2 + OGD/R + Corilagin and siAbcg2 + OGD/R + Corilagin groups than in the siNC + OGD/R + Corilagin group, with P values ranging from <0.001 to 0.02 (Figures 9F,G). These findings support the involvement of NRF2 and ABCG2 in the preservation of tight junction protein expression associated with Corilagin treatment under OGD/R conditions.

## 4 Discussion

IIRI is a complex pathological process involving ischemia, reperfusion, oxidative stress, inflammation, ERS, and cell death (Shi et al., 2024; Zhao et al., 2024; Zou and Wang, 2022; Mi et al., 2021; Alicehajic et al., 2024; Wang et al., 2024b; Kip et al., 2022). Despite continued advances, current clinical management emphasizes early diagnosis, prompt restoration of blood flow, and supportive care (Bala et al., 2022; Theodore et al., 2024), and targeted pharmacotherapies for IIRI remain insufficiently established (Wang et al., 2024b). Corilagin has antioxidant, anti-inflammatory, anti-apoptotic, and cell stress-modulating activities and can alleviate IIRI-induced injury in the intestine and distant organs (Wang et al., 2023a; Li et al., 2022; Li et al., 2023; Wang et al., 2024a; Wang et al., 2023b). Previous studies have attributed its protection to AMPK/Sirt1-mediated autophagy, the NLRP3 inflammasome and pyroptosis, BiP-related ERS, and NCOA4-mediated ferroptosis (Li et al., 2022; Li et al., 2023; Wang et al., 2024a; Wang et al., 2023b; Lee, 2005). Building on this work, we integrated human and mouse transcriptomic data, machine-learning screening, molecular dynamics simulations, and *in vivo* and *in vitro* experiments. Corilagin ameliorated IIRI-induced intestinal mucosal injury, reduced inflammatory and cellular stress responses, and restored the expression of intestinal epithelial barrier proteins. Further analyses identified ABCG2 as an important candidate molecule in Corilagin-mediated barrier protection and suggested that NRF2 may participate in its upstream regulation. These findings provide a new direction for understanding the multitarget protective effects of Corilagin. However, the current evidence should be considered a mechanistic clue rather than proof of a fully established direct pathway.

We first used cross-species transcriptomic integration and machine-learning methods to screen candidate genes associated with IIRI. IL10, C5AR1, SELE, HMOX1, ABCG2, ABCC1, AXL, ABL2, FGF2, and PRKCZ were linked to inflammation, oxidative stress, cell adhesion, substrate transport, and tissue repair. NRF2 was not among the 12 candidate core genes identified by machine learning. It should therefore not be described as a core target directly selected by the machine-learning analysis. The subsequent investigation of NRF2 was motivated by the prominent changes in ABCG2 observed here and by the established roles of NRF2 in oxidative-stress responses and intestinal-barrier homeostasis (Zhang et al., 2023c; Petsouki et al., 2022; Cuadrado et al., 2019). Evidence concerning ABCG2 function in the intestinal epithelial barrier (Shi et al., 2025) and direct NRF2-dependent regulation of ABCG2 in cancer-cell models (Singh et al., 2010) provided additional rationale. Accordingly, machine learning first implicated ABCG2 in IIRI and Corilagin-mediated protection. Literature evidence and experimental findings were then used to explore a potential upstream regulator. The NRF2-ABCG2 relationship was not inferred directly from the machine-learning results. Moreover, the models were developed using a relatively limited sample size. Although cross-validation and multiple algorithms were used, the algorithms shared the same training data. Five-fold cross-validation primarily provided internal model assessment and hyperparameter optimization. It cannot replace validation in an independent cohort or fully exclude overfitting (Greener et al., 2022; Vabalas et al., 2019). The 12 genes are therefore more appropriately described as candidate core genes prioritized by machine learning. Their robustness and translational relevance require validation in larger independent cohorts and functional experiments.

ABCG2 is an important member of the ATP-binding cassette transporter family. In intestinal epithelial cells, it localizes to the apical membrane and transports diverse endogenous and exogenous substrates. Through these functions, ABCG2 contributes to the material barrier between the intestinal lumen and tissues and to local homeostasis (Shi et al., 2025). During IIRI, oxidative stress, inflammation, and epithelial injury disrupt tight junctions and increase permeability (Wang et al., 2025; Zhang et al., 2023c; Wang et al., 2024b), changes that may also impair epithelial transport functions (Shi et al., 2025). ABCG2 expression decreased in our IIRI model, whereas Corilagin partially restored its expression. Similar changes were observed in the OGD/R cell model. Abcg2 knockdown markedly weakened the effects of Corilagin on cellular injury, inflammation, and barrier-related indicators, suggesting that ABCG2 contributes to its cytoprotective action. ABCC1 expression also changed after Corilagin treatment in the sham group. Corilagin may therefore affect multiple ABC transporters, whose responses to injury and treatment may differ. The ABCC1 change cannot be interpreted simply as reversal of an IIRI-induced abnormality. It may reflect the combined effects of direct drug induction and injury-related regulation. Overall, our findings more strongly support ABCG2 as a candidate molecule associated with both IIRI severity and Corilagin-mediated protection. Whether ABCG2 directly determines barrier recovery requires ABCG2 overexpression, *in vivo* genetic interventions, intestinal permeability assays, and transepithelial electrical resistance measurements.

NRF2 is a central transcription factor in cellular antioxidant-stress responses (Sengoku et al., 2022). Its activity is regulated mainly through protein stability, KEAP1-mediated degradation, nuclear translocation, and post-translational mechanisms; functional activity can therefore change without a corresponding change in transcript abundance (Cuadrado et al., 2019). NRF2 has been shown to regulate ABCG2 through an ARE in the ABCG2 promoter in cancer-cell models (Singh et al., 2010). We found that Corilagin promoted NRF2 nuclear translocation and increased ABCG2 expression. Nrf2 knockdown also weakened the protective effects of Corilagin against cellular injury. These findings suggest that NRF2 may participate in the ABCG2-related protective process. However, direct transcriptional regulation of ABCG2 by NRF2 in our model was not established using promoter-reporter assays, chromatin immunoprecipitation, or binding-site mutagenesis. Thus, the evidence supports the wording that NRF2 may participate in ABCG2-related regulation, rather than establishing a definitive NRF2-ABCG2 axis. Nrf2 knockdown may also affect multiple antioxidant and cytoprotective genes. The current results therefore cannot exclude protection mediated through other NRF2 targets. ABCG2 promoter assays, ChIP-qPCR, ARE-reporter analyses, bidirectional NRF2 and ABCG2 perturbation, and rescue experiments are needed to define causality.

Molecular dynamics simulations and binding free-energy analysis provided structural evidence for a potential spatial association between Corilagin and NRF2. The overall RMSD increased during the simulation and approached a plateau at a higher level, suggesting substantial protein conformational changes. Global RMSD summarizes the overall deviation of the protein or complex from its initial structure; an increase alone is insufficient to establish ligand dissociation or irreversible protein unfolding. In response to the reviewer’s concern regarding the atypical RMSD, we re-examined trajectory processing and periodic boundary conditions. We also added local ligand-motion analyses after alignment to the protein backbone and binding region. Corilagin remained spatially close to the predicted binding region during the simulated interval and formed hydrogen-bond and hydrophobic interactions. The global RMSD change may therefore partly reflect overall protein conformational adjustment and cannot be equated with complete ligand dissociation. Nevertheless, local proximity does not establish stable binding or direct binding affinity. MM/GBSA is an end-state free-energy estimation method based on sampled MD trajectories; the present estimate should therefore not be treated as a direct measurement of binding affinity (Valdés-Tresanco et al., 2021). A single trajectory of limited duration also cannot demonstrate complete convergence (Hollingsworth and Dror, 2018). Accordingly, these simulations provide computational evidence of a potential local interaction between Corilagin and NRF2, rather than independent proof of a stable complex. Multiple independent trajectories, longer simulations, and direct binding assays such as surface plasmon resonance or microscale thermophoresis are required.

Several limitations require further consideration. First, the human and mouse transcriptomic datasets were generated using different experimental systems and time points. The human data mainly captured changes after 30 and 120 min of reperfusion, whereas the mouse data covered 3 and 6 h. Our animal experiments used 45 min of ischemia followed by 1 h of reperfusion. These time points do not necessarily represent the same pathological stage (Zong et al., 2023; Kip et al., 2022; Breschi et al., 2017). Thus, shared cross-species genes may reflect relatively conserved biological changes during IIRI. They do not imply identical expression patterns at every time point. Second, the machine-learning candidates require validation in independent clinical samples, with tissue localization, protein-level measurements, and functional perturbation (Greener et al., 2022; Vabalas et al., 2019). Third, this study focused mainly on NRF2, ABCG2, and intestinal epithelial barrier-related indicators. We did not systematically characterize inflammatory-cell infiltration, immune-cell states, redox metabolism, or other transporters. Finally, although Abcg2 knockdown supports its involvement in Corilagin-mediated protection, it does not establish ABCG2 as the sole or decisive target. Corilagin may improve IIRI through interconnected effects on ERS, oxidative stress, inflammation, autophagy, and cell death (Li et al., 2022; Li et al., 2023; Wang et al., 2024a; Wang et al., 2023b). ABCG2 may be one important node linking cellular homeostasis to barrier function.

In conclusion, we integrated transcriptomics, machine learning, structural simulations, and *in vivo* and *in vitro* experiments. Our findings suggest that Corilagin may alleviate intestinal epithelial barrier injury in IIRI by modulating NRF2-related stress responses and promoting ABCG2 expression. Compared with previous studies of AMPK/Sirt1 signaling, autophagy, the NLRP3 inflammasome, and BiP-mediated ERS (Li et al., 2022; Li et al., 2023; Wang et al., 2024a), our work further highlights a potential link between ABC transporters and antioxidant stress regulation. This provides a new framework for understanding the multilayered protective mechanisms of Corilagin. However, the machine-learning screen, molecular dynamics simulations, and proposed causal relationship between NRF2 and ABCG2 remain subject to important limitations. Independent samples, direct binding assays, promoter-regulation studies, and *in vivo* genetic interventions are required to establish the underlying molecular mechanism.

## 5 Conclusion

This study provides an integrative framework for understanding the multitarget protective effects of Corilagin in intestinal ischemia–reperfusion injury. Rather than acting through a single molecular target, Corilagin appears to influence interconnected processes involving stress responses, epithelial homeostasis, and barrier protection. ABCG2 emerges as a biologically relevant candidate linking epithelial transport function with intestinal barrier preservation, while NRF2-related stress signaling may contribute to its regulation. Collectively, the computational and experimental evidence supports a potential Corilagin–NRF2/ABCG2 regulatory relationship associated with attenuation of intestinal injury and modulation of ERS-related responses, thereby providing a mechanistic basis for further investigation of Corilagin as a potential therapeutic candidate for IIRI.

## Data Availability

The datasets presented in this study can be found in online repositories. The names of the repository/repositories and accession number(s) can be found in the article/Supplementary Material.
