# Supplementary material for: Multi-source data integration and ensemble machine learning analysis of the role of Corilagin in regulating endoplasmic reticulum stress and alleviating intestinal ischemia reperfusion injury in mice
Source: Front Pharmacol. 2026 Oct 9;17:1966534. doi: 10.3389/fphar.2026.1966534 (PMC13691303; doi:10.3389/fphar.2026.1966534)
Supplement: Supplementary file 1 [file Supplementaryfile6.pdf]

S1

Model	DatasetA (train set)	DatasetB (test set)	Average of test set
NaiveBayes+Lasso−CV:5 fold	0.839	0.941	0.941
NN−MLP (cutoff:0.25, lr:0.001, bs:5, ep:50, dropout:0.25)	0.800	0.800	0.8
NN−MLP (cutoff:0.5, lr:0.001, bs:5, ep:50, dropout:0.25)	0.800	0.800	0.8
NN−MLP (cutoff:0.25, lr:0.001, bs:5, ep:50, dropout:0.5)	0.800	0.800	0.8
NN−MLP (cutoff:0.5, lr:0.001, bs:5, ep:50, dropout:0.5)	0.800	0.800	0.8
NN−MLP (cutoff:0.25, lr:0.001, bs:5, ep:50, dropout:0.75)	0.800	0.800	0.8
NN−MLP (cutoff:0.25, lr:0.005, bs:5, ep:50, dropout:0.25)	0.800	0.800	0.8
NN−MLP (cutoff:0.5, lr:0.005, bs:5, ep:50, dropout:0.25)	0.800	0.800	0.8
NN−MLP (cutoff:0.25, lr:0.005, bs:5, ep:50, dropout:0.5)	0.800	0.800	0.8
NN−MLP (cutoff:0.5, lr:0.005, bs:5, ep:50, dropout:0.5)	0.800	0.800	0.8
NN−MLP (cutoff:0.25, lr:0.005, bs:5, ep:50, dropout:0.75)	0.800	0.800	0.8
NN−MLP (cutoff:0.5, lr:0.005, bs:5, ep:50, dropout:0.75)	0.800	0.800	0.8
NN−MLP (cutoff:0.25, lr:0.01, bs:5, ep:50, dropout:0.25)	0.800	0.800	0.8
NN−MLP (cutoff:0.5, lr:0.01, bs:5, ep:50, dropout:0.25)	0.800	0.800	0.8
NN−MLP (cutoff:0.25, lr:0.01, bs:5, ep:50, dropout:0.5)	0.800	0.800	0.8
NN−MLP (cutoff:0.5, lr:0.01, bs:5, ep:50, dropout:0.5)	0.800	0.800	0.8
NN−MLP (cutoff:0.25, lr:0.01, bs:5, ep:50, dropout:0.75)	0.800	0.800	0.8
NN−MLP (cutoff:0.5, lr:0.01, bs:5, ep:50, dropout:0.75)	0.800	0.800	0.8
NN−MLP (cutoff:0.25, lr:0.05, bs:5, ep:50, dropout:0.25)	0.800	0.800	0.8
NN−MLP (cutoff:0.5, lr:0.05, bs:5, ep:50, dropout:0.25)	0.800	0.800	0.8
NN−MLP (cutoff:0.25, lr:0.05, bs:5, ep:50, dropout:0.5)	0.800	0.800	0.8
NN−MLP (cutoff:0.5, lr:0.05, bs:5, ep:50, dropout:0.5)	0.800	0.800	0.8
NN−MLP (cutoff:0.25, lr:0.05, bs:5, ep:50, dropout:0.75)	0.800	0.800	0.8
NN−MLP (cutoff:0.5, lr:0.05, bs:5, ep:50, dropout:0.75)	0.800	0.800	0.8
NN−MLP (cutoff:0.25, lr:0.001, bs:15, ep:50, dropout:0.25)	0.800	0.800	0.8
NN−MLP (cutoff:0.5, lr:0.001, bs:15, ep:50, dropout:0.25)	0.800	0.800	0.8
NN−MLP (cutoff:0.25, lr:0.001, bs:15, ep:50, dropout:0.5)	0.800	0.800	0.8
NN−MLP (cutoff:0.5, lr:0.001, bs:15, ep:50, dropout:0.5)	0.800	0.800	0.8
NN−MLP (cutoff:0.25, lr:0.001, bs:15, ep:50, dropout:0.75)	0.800	0.800	0.8
NN−MLP (cutoff:0.25, lr:0.005, bs:15, ep:50, dropout:0.25)	0.800	0.800	0.8
NN−MLP (cutoff:0.5, lr:0.005, bs:15, ep:50, dropout:0.25)	0.800	0.800	0.8
NN−MLP (cutoff:0.25, lr:0.005, bs:15, ep:50, dropout:0.5)	0.800	0.800	0.8
NN−MLP (cutoff:0.5, lr:0.005, bs:15, ep:50, dropout:0.5)	0.800	0.800	0.8
NN−MLP (cutoff:0.25, lr:0.005, bs:15, ep:50, dropout:0.75)	0.800	0.800	0.8
NN−MLP (cutoff:0.5, lr:0.005, bs:15, ep:50, dropout:0.75)	0.800	0.800	0.8
NN−MLP (cutoff:0.25, lr:0.01, bs:15, ep:50, dropout:0.25)	0.800	0.800	0.8
NN−MLP (cutoff:0.5, lr:0.01, bs:15, ep:50, dropout:0.25)	0.800	0.800	0.8
NN−MLP (cutoff:0.25, lr:0.01, bs:15, ep:50, dropout:0.5)	0.800	0.800	0.8
NN−MLP (cutoff:0.5, lr:0.01, bs:15, ep:50, dropout:0.5)	0.800	0.800	0.8
NN−MLP (cutoff:0.25, lr:0.01, bs:15, ep:50, dropout:0.75)	0.800	0.800	0.8
NN−MLP (cutoff:0.5, lr:0.01, bs:15, ep:50, dropout:0.75)	0.800	0.800	0.8
NN−MLP (cutoff:0.25, lr:0.05, bs:15, ep:50, dropout:0.25)	0.800	0.800	0.8
NN−MLP (cutoff:0.5, lr:0.05, bs:15, ep:50, dropout:0.25)	0.800	0.800	0.8
NN−MLP (cutoff:0.25, lr:0.05, bs:15, ep:50, dropout:0.5)	0.800	0.800	0.8
NN−MLP (cutoff:0.5, lr:0.05, bs:15, ep:50, dropout:0.5)	0.800	0.800	0.8
NN−MLP (cutoff:0.25, lr:0.05, bs:15, ep:50, dropout:0.75)	0.800	0.800	0.8
NN−MLP (cutoff:0.5, lr:0.05, bs:15, ep:50, dropout:0.75)	0.800	0.800	0.8
LR (cutoff:0.25)	1.000	0.800	0.8
LR (cutoff:0.5)	1.000	0.800	0.8
LR (cutoff:0.75)	1.000	0.800	0.8
QDA+Lasso−CV:5 fold	0.733	0.800	0.8
KNN+Lasso−CV:5 fold (k=1)	1.000	0.800	0.8
KNN+Lasso−CV:5 fold (k=2)	0.963	0.800	0.8
KNN+Lasso−CV:5 fold (k=3)	1.000	0.800	0.8
KNN+Lasso−CV:5 fold (k=4)	0.966	0.800	0.8
KNN+Lasso−CV:5 fold (k=5)	0.933	0.800	0.8
DT	1.000	0.800	0.8
RF (mtry=18, 25%p)	1.000	0.800	0.8
RF (mtry=34, 50%p)	0.889	0.800	0.8
XGBoost−default (cutoff:0.25)	1.000	0.800	0.8
XGBoost−default (cutoff:0.5)	1.000	0.800	0.8
XGBoost−default (cutoff:0.75)	1.000	0.800	0.8
XGBoost−CV:5 fold (cutoff:0.25)	1.000	0.800	0.8
XGBoost−CV:5 fold (cutoff:0.5)	1.000	0.800	0.8
XGBoost−CV:5 fold (cutoff:0.75)	1.000	0.800	0.8
SVM−default (kernel: radial)	1.000	0.800	0.8
SVM−default+Lasso−CV:5 fold (kernel: linear)	0.800	0.800	0.8
SVM−default+Lasso−CV:5 fold (kernel: radial)	0.800	0.800	0.8
SVM−CV:5 fold (kernel: radial)	0.800	0.800	0.8
SVM−CV:5 fold+Lasso−CV:5 fold (kernel: linear)	0.800	0.800	0.8
SVM−CV:5 fold+Lasso−CV:5 fold (kernel: radial)	0.800	0.800	0.8
SVM−CV:5 fold+Lasso−CV:5 fold (kernel: polynomial)	0.800	0.800	0.8
GBM−default (cutoff:0.25)	1.000	0.800	0.8
GBM−default (cutoff:0.5)	1.000	0.800	0.8
GBM−default (cutoff:0.75)	1.000	0.800	0.8
GBM−CV:5 fold (cutoff:0.25)	0.966	0.800	0.8
GBM−CV:5 fold (cutoff:0.5)	1.000	0.800	0.8
GBM−CV:5 fold (cutoff:0.75)	1.000	0.800	0.8
GBM−default+Lasso−CV:5 fold (cutoff:0.25)	0.848	0.800	0.8
StepWise−AIC+LR (cutoff:0.25)	1.000	0.800	0.8
StepWise−AIC+LR (cutoff:0.5)	1.000	0.800	0.8
StepWise−AIC+LR (cutoff:0.75)	1.000	0.800	0.8
NaiveBayes	1.000	0.706	0.706
NN−MLP (cutoff:0.5, lr:0.001, bs:15, ep:50, dropout:0.75)	0.800	0.571	0.571
KNN (k=2)	0.846	0.571	0.571
LDA	1.000	0.500	0.5
KNN (k=1)	1.000	0.364	0.364
NN−MLP (cutoff:0.5, lr:0.001, bs:5, ep:50, dropout:0.75)	0.800	0.222	0.222
KNN (k=3)	0.889	0.200	0.2
KNN (k=4)	0.828	0.182	0.182
RF+Lasso−CV:5 fold (mtry=34, 50%p)	1.000	0.000	0
RF+Lasso−CV:5 fold (mtry=50, 75%p)	1.000	0.000	0
Lasso.R−CV:5 fold (cutoff:0.25)	1.000	0.000	0
Lasso.R−CV:5 fold (cutoff:0.5)	1.000	0.000	0
Lasso.R−CV:5 fold (cutoff:0.75)	1.000	0.000	0
ENR−CV:5 fold (cutoff:0.25, alpha:0.1)	1.000	0.000	0
ENR−CV:5 fold (cutoff:0.5, alpha:0.1)	1.000	0.000	0
ENR−CV:5 fold (cutoff:0.75, alpha:0.1)	1.000	0.000	0
ENR−CV:5 fold (cutoff:0.25, alpha:0.2)	1.000	0.000	0
ENR−CV:5 fold (cutoff:0.5, alpha:0.2)	1.000	0.000	0
ENR−CV:5 fold (cutoff:0.75, alpha:0.2)	1.000	0.000	0
ENR−CV:5 fold (cutoff:0.25, alpha:0.3)	1.000	0.000	0
ENR−CV:5 fold (cutoff:0.5, alpha:0.3)	1.000	0.000	0
ENR−CV:5 fold (cutoff:0.75, alpha:0.3)	1.000	0.000	0
ENR−CV:5 fold (cutoff:0.25, alpha:0.4)	1.000	0.000	0
ENR−CV:5 fold (cutoff:0.5, alpha:0.4)	1.000	0.000	0
ENR−CV:5 fold (cutoff:0.75, alpha:0.4)	1.000	0.000	0
ENR−CV:5 fold (cutoff:0.25, alpha:0.5)	1.000	0.000	0
ENR−CV:5 fold (cutoff:0.5, alpha:0.5)	1.000	0.000	0
ENR−CV:5 fold (cutoff:0.75, alpha:0.5)	1.000	0.000	0
ENR−CV:5 fold (cutoff:0.25, alpha:0.6)	1.000	0.000	0
ENR−CV:5 fold (cutoff:0.5, alpha:0.6)	1.000	0.000	0
ENR−CV:5 fold (cutoff:0.75, alpha:0.6)	1.000	0.000	0
ENR−CV:5 fold (cutoff:0.25, alpha:0.7)	1.000	0.000	0
ENR−CV:5 fold (cutoff:0.5, alpha:0.7)	1.000	0.000	0
ENR−CV:5 fold (cutoff:0.75, alpha:0.7)	1.000	0.000	0
ENR−CV:5 fold (cutoff:0.25, alpha:0.8)	1.000	0.000	0
ENR−CV:5 fold (cutoff:0.5, alpha:0.8)	1.000	0.000	0
ENR−CV:5 fold (cutoff:0.75, alpha:0.8)	1.000	0.000	0
ENR−CV:5 fold (cutoff:0.25, alpha:0.9)	1.000	0.000	0
ENR−CV:5 fold (cutoff:0.5, alpha:0.9)	1.000	0.000	0
ENR−CV:5 fold (cutoff:0.75, alpha:0.9)	1.000	0.000	0
SVM−default (kernel: linear)	1.000	0.000	0
SVM−CV:5 fold (kernel: linear)	1.000	0.000	0
SVM−CV:5 fold (kernel: polynomial)	1.000	0.000	0
GBM−CV:5 fold+Lasso−CV:5 fold (cutoff:0.25)	1.000	0.000	0
GBM−CV:5 fold+Lasso−CV:5 fold (cutoff:0.5)	1.000	0.000	0
GBM−CV:5 fold+Lasso−CV:5 fold (cutoff:0.75)	1.000	0.000	0

Average of test set (axis: 0, 0.5, 1)

F−score: 1, 0.5, 0

Data set: DatasetA (train set), DatasetB (test set)

S2

Model	DatasetB (train set)	DatasetA (test set)	Average of test set
NN−MLP (cutoff:0.25, lr:0.001, bs:5, ep:50, dropout:0.25)	0.842	0.800	0.8
NN−MLP (cutoff:0.25, lr:0.001, bs:5, ep:50, dropout:0.5)	0.800	0.800	0.8
NN−MLP (cutoff:0.5, lr:0.001, bs:5, ep:50, dropout:0.5)	0.800	0.800	0.8
NN−MLP (cutoff:0.25, lr:0.001, bs:5, ep:50, dropout:0.75)	0.800	0.800	0.8
NN−MLP (cutoff:0.5, lr:0.001, bs:5, ep:50, dropout:0.75)	0.800	0.800	0.8
NN−MLP (cutoff:0.25, lr:0.005, bs:5, ep:50, dropout:0.25)	0.800	0.800	0.8
NN−MLP (cutoff:0.5, lr:0.005, bs:5, ep:50, dropout:0.25)	0.800	0.800	0.8
NN−MLP (cutoff:0.25, lr:0.005, bs:5, ep:50, dropout:0.5)	0.800	0.800	0.8
NN−MLP (cutoff:0.5, lr:0.005, bs:5, ep:50, dropout:0.5)	0.800	0.800	0.8
NN−MLP (cutoff:0.25, lr:0.005, bs:5, ep:50, dropout:0.75)	0.800	0.800	0.8
NN−MLP (cutoff:0.5, lr:0.005, bs:5, ep:50, dropout:0.75)	0.800	0.800	0.8
NN−MLP (cutoff:0.25, lr:0.01, bs:5, ep:50, dropout:0.25)	0.800	0.800	0.8
NN−MLP (cutoff:0.5, lr:0.01, bs:5, ep:50, dropout:0.25)	0.800	0.800	0.8
NN−MLP (cutoff:0.25, lr:0.01, bs:5, ep:50, dropout:0.5)	0.800	0.800	0.8
NN−MLP (cutoff:0.5, lr:0.01, bs:5, ep:50, dropout:0.5)	0.800	0.800	0.8
NN−MLP (cutoff:0.25, lr:0.01, bs:5, ep:50, dropout:0.75)	0.800	0.800	0.8
NN−MLP (cutoff:0.5, lr:0.01, bs:5, ep:50, dropout:0.75)	0.800	0.800	0.8
NN−MLP (cutoff:0.25, lr:0.05, bs:5, ep:50, dropout:0.25)	0.800	0.800	0.8
NN−MLP (cutoff:0.5, lr:0.05, bs:5, ep:50, dropout:0.25)	0.800	0.800	0.8
NN−MLP (cutoff:0.25, lr:0.05, bs:5, ep:50, dropout:0.5)	0.800	0.800	0.8
NN−MLP (cutoff:0.5, lr:0.05, bs:5, ep:50, dropout:0.5)	0.800	0.800	0.8
NN−MLP (cutoff:0.25, lr:0.05, bs:5, ep:50, dropout:0.75)	0.800	0.800	0.8
NN−MLP (cutoff:0.5, lr:0.05, bs:5, ep:50, dropout:0.75)	0.800	0.800	0.8
NN−MLP (cutoff:0.75, lr:0.05, bs:5, ep:50, dropout:0.75)	0.800	0.800	0.8
LDA+Lasso−CV:5 fold	0.778	0.800	0.8
QDA	0.778	0.800	0.8
QDA+Lasso−CV:5 fold	0.857	0.800	0.8
KNN (k=1)	1.000	0.800	0.8
KNN (k=2)	0.800	0.800	0.8
KNN (k=3)	0.769	0.800	0.8
DT	0.933	0.800	0.8
DT+Lasso−CV:5 fold	0.933	0.800	0.8
RF+Lasso−CV:5 fold (mtry=18, 25%p)	0.933	0.800	0.8
RF+Lasso−CV:5 fold (mtry=34, 50%p)	0.875	0.800	0.8
RF+Lasso−CV:5 fold (mtry=50, 75%p)	0.875	0.800	0.8
XGBoost−default (cutoff:0.25)	0.800	0.800	0.8
XGBoost−default (cutoff:0.5)	1.000	0.800	0.8
XGBoost−default+Lasso−CV:5 fold (cutoff:0.25)	0.800	0.800	0.8
XGBoost−default+Lasso−CV:5 fold (cutoff:0.5)	0.933	0.800	0.8
XGBoost−default+Lasso−CV:5 fold (cutoff:0.75)	0.933	0.800	0.8
XGBoost−CV:5 fold (cutoff:0.5)	1.000	0.800	0.8
XGBoost−CV:5 fold (cutoff:0.75)	0.800	0.800	0.8
XGBoost−CV:5 fold+Lasso−CV:5 fold (cutoff:0.25)	0.933	0.800	0.8
XGBoost−CV:5 fold+Lasso−CV:5 fold (cutoff:0.5)	0.933	0.800	0.8
XGBoost−CV:5 fold+Lasso−CV:5 fold (cutoff:0.75)	0.800	0.800	0.8
SVM−default (kernel: radial)	1.000	0.800	0.8
SVM−default+Lasso−CV:5 fold (kernel: linear)	0.933	0.800	0.8
SVM−default+Lasso−CV:5 fold (kernel: polynomial)	0.800	0.800	0.8
SVM−default+Lasso−CV:5 fold (kernel: radial)	0.778	0.800	0.8
SVM−CV:5 fold (kernel: radial)	1.000	0.800	0.8
SVM−CV:5 fold+Lasso−CV:5 fold (kernel: linear)	0.933	0.800	0.8
SVM−CV:5 fold+Lasso−CV:5 fold (kernel: radial)	0.857	0.800	0.8
SVM−CV:5 fold+Lasso−CV:5 fold (kernel: polynomial)	0.800	0.800	0.8
GBM−default (cutoff:0.25)	1.000	0.800	0.8
GBM−CV:5 fold (cutoff:0.25)	1.000	0.800	0.8
GBM−default+Lasso−CV:5 fold (cutoff:0.25)	0.933	0.800	0.8
GBM−default+Lasso−CV:5 fold (cutoff:0.5)	0.933	0.800	0.8
GBM−default+Lasso−CV:5 fold (cutoff:0.75)	0.933	0.800	0.8
GBM−CV:5 fold+Lasso−CV:5 fold (cutoff:0.25)	0.933	0.800	0.8
GBM−CV:5 fold+Lasso−CV:5 fold (cutoff:0.5)	0.933	0.800	0.8
GBM−CV:5 fold+Lasso−CV:5 fold (cutoff:0.75)	0.933	0.800	0.8
KNN (k=4)	0.857	0.765	0.765
KNN (k=5)	0.769	0.765	0.765
NN−MLP (cutoff:0.5, lr:0.001, bs:5, ep:50, dropout:0.25)	1.000	0.552	0.552
NaiveBayes+Lasso−CV:5 fold	0.857	0.545	0.545
RF (mtry=18, 25%p)	1.000	0.522	0.522
NaiveBayes	1.000	0.476	0.476
LDA	1.000	0.417	0.417
LR (cutoff:0.25)	1.000	0.000	
LR (cutoff:0.5)	1.000	0.000	
LR (cutoff:0.75)	1.000	0.000	
KNN+Lasso−CV:5 fold (k=1)	1.000	0.000	
KNN+Lasso−CV:5 fold (k=2)	1.000	0.000	
KNN+Lasso−CV:5 fold (k=3)	1.000	0.000	
KNN+Lasso−CV:5 fold (k=4)	1.000	0.000	
KNN+Lasso−CV:5 fold (k=5)	1.000	0.000	
RF (mtry=34, 50%p)	1.000	0.000	
RF (mtry=50, 75%p)	1.000	0.000	
XGBoost−default (cutoff:0.75)	1.000	0.000	
RR−CV:5 fold (cutoff:0.25)	1.000	0.000	
RR−CV:5 fold (cutoff:0.5)	1.000	0.000	
RR−CV:5 fold (cutoff:0.75)	1.000	0.000	
Lasso.R−CV:5 fold (cutoff:0.25)	1.000	0.000	
Lasso.R−CV:5 fold (cutoff:0.5)	1.000	0.000	
Lasso.R−CV:5 fold (cutoff:0.75)	1.000	0.000	
ENR−CV:5 fold (cutoff:0.25, alpha:0.1)	1.000	0.000	
ENR−CV:5 fold (cutoff:0.5, alpha:0.1)	1.000	0.000	
ENR−CV:5 fold (cutoff:0.75, alpha:0.1)	1.000	0.000	
ENR−CV:5 fold (cutoff:0.25, alpha:0.2)	1.000	0.000	
ENR−CV:5 fold (cutoff:0.5, alpha:0.2)	1.000	0.000	
ENR−CV:5 fold (cutoff:0.75, alpha:0.2)	1.000	0.000	
ENR−CV:5 fold (cutoff:0.25, alpha:0.3)	1.000	0.000	
ENR−CV:5 fold (cutoff:0.5, alpha:0.3)	1.000	0.000	
ENR−CV:5 fold (cutoff:0.75, alpha:0.3)	1.000	0.000	
ENR−CV:5 fold (cutoff:0.25, alpha:0.4)	1.000	0.000	
ENR−CV:5 fold (cutoff:0.5, alpha:0.4)	1.000	0.000	
ENR−CV:5 fold (cutoff:0.75, alpha:0.4)	1.000	0.000	
ENR−CV:5 fold (cutoff:0.25, alpha:0.5)	1.000	0.000	
ENR−CV:5 fold (cutoff:0.5, alpha:0.5)	1.000	0.000	
ENR−CV:5 fold (cutoff:0.75, alpha:0.5)	1.000	0.000	
SVM−default (kernel: linear)	1.000	0.000	
SVM−default (kernel: polynomial)	1.000	0.000	
SVM−CV:5 fold (kernel: linear)	1.000	0.000	
SVM−CV:5 fold (kernel: polynomial)	1.000	0.000	
GBM−default (cutoff:0.5)	1.000	0.000	
GBM−default (cutoff:0.75)	1.000	0.000	
GBM−CV:5 fold (cutoff:0.5)	1.000	0.000	
GBM−CV:5 fold (cutoff:0.75)	1.000	0.000	
StepWise−AIC+LR (cutoff:0.25)	1.000	0.000	
StepWise−AIC+LR (cutoff:0.5)	1.000	0.000	
StepWise−AIC+LR (cutoff:0.75)	1.000	0.000	

F−score: 1, 0.5, 0

Data set: DatasetA (test set), DatasetB (train set)

0 0.2 0.4 0.6 0.8

Average of test set

S3

Method	DatasetA (train set)	DatasetB (test set)	DatasetC (test set)	DatasetD (test set)	Average of test set
NaiveBayes+Lasso−CV:5 fold	1.000	0.833	0.889	0.889	0.87
KNN+Lasso−CV:5 fold (k=1)	1.000	1.000	0.667	0.667	0.778
KNN+Lasso−CV:5 fold (k=2)	0.923	1.000	0.667	0.667	0.778
KNN+Lasso−CV:5 fold (k=3)	1.000	1.000	0.667	0.667	0.778
KNN+Lasso−CV:5 fold (k=4)	1.000	1.000	0.667	0.667	0.778
KNN (k=1)	1.000	1.000	0.667	0.667	0.778
KNN (k=2)	1.000	1.000	0.667	0.667	0.778
DT	1.000	1.000	0.667	0.667	0.778
DT+Lasso−CV:5 fold	1.000	1.000	0.667	0.667	0.778
RF (mtry=18, 25%p)	1.000	1.000	0.667	0.667	0.778
RF (mtry=34, 50%p)	1.000	1.000	0.667	0.667	0.778
RF (mtry=50, 75%p)	1.000	1.000	0.667	0.667	0.778
RF+Lasso−CV:5 fold (mtry=18, 25%p)	0.923	1.000	0.667	0.667	0.778
RF+Lasso−CV:5 fold (mtry=50, 75%p)	1.000	1.000	0.667	0.667	0.778
XGBoost−default (cutoff:0.25)	1.000	1.000	0.667	0.667	0.778
XGBoost−default (cutoff:0.5)	1.000	1.000	0.667	0.667	0.778
XGBoost−default (cutoff:0.75)	1.000	1.000	0.667	0.667	0.778
XGBoost−default+Lasso−CV:5 fold (cutoff:0.25)	1.000	1.000	0.667	0.667	0.778
XGBoost−default+Lasso−CV:5 fold (cutoff:0.5)	1.000	1.000	0.667	0.667	0.778
XGBoost−default+Lasso−CV:5 fold (cutoff:0.75)	1.000	1.000	0.667	0.667	0.778
XGBoost−CV:5 fold (cutoff:0.25)	1.000	1.000	0.667	0.667	0.778
XGBoost−CV:5 fold (cutoff:0.5)	1.000	1.000	0.667	0.667	0.778
XGBoost−CV:5 fold (cutoff:0.75)	1.000	1.000	0.667	0.667	0.778
XGBoost−CV:5 fold+Lasso−CV:5 fold (cutoff:0.25)	1.000	1.000	0.667	0.667	0.778
XGBoost−CV:5 fold+Lasso−CV:5 fold (cutoff:0.5)	1.000	1.000	0.667	0.667	0.778
XGBoost−CV:5 fold+Lasso−CV:5 fold (cutoff:0.75)	1.000	1.000	0.667	0.667	0.778
GBM−default (cutoff:0.25)	1.000	1.000	0.667	0.667	0.778
GBM−default (cutoff:0.5)	1.000	1.000	0.667	0.667	0.778
GBM−default (cutoff:0.75)	1.000	1.000	0.667	0.667	0.778
GBM−CV:5 fold (cutoff:0.25)	1.000	1.000	0.667	0.667	0.778
GBM−CV:5 fold (cutoff:0.5)	1.000	1.000	0.667	0.667	0.778
GBM−CV:5 fold (cutoff:0.75)	1.000	1.000	0.667	0.667	0.778
GBM−default+Lasso−CV:5 fold (cutoff:0.25)	1.000	1.000	0.667	0.667	0.778
GBM−default+Lasso−CV:5 fold (cutoff:0.5)	1.000	1.000	0.667	0.667	0.778
GBM−default+Lasso−CV:5 fold (cutoff:0.75)	1.000	1.000	0.667	0.667	0.778
GBM−CV:5 fold+Lasso−CV:5 fold (cutoff:0.25)	1.000	1.000	0.667	0.667	0.778
GBM−CV:5 fold+Lasso−CV:5 fold (cutoff:0.5)	1.000	1.000	0.667	0.667	0.778
GBM−CV:5 fold+Lasso−CV:5 fold (cutoff:0.75)	1.000	1.000	0.667	0.667	0.778
StepWise−AIC+LR (cutoff:0.25)	1.000	1.000	0.667	0.667	0.778
StepWise−AIC+LR (cutoff:0.5)	1.000	1.000	0.667	0.667	0.778
StepWise−AIC+LR (cutoff:0.75)	1.000	1.000	0.667	0.667	0.778
LDA	0.615	0.875	0.727	0.727	0.777
KNN (k=3)	1.000	0.923	0.667	0.667	0.752
KNN (k=4)	1.000	0.923	0.667	0.667	0.752
KNN (k=5)	1.000	0.923	0.667	0.667	0.752
SVM−default+Lasso−CV:5 fold (kernel: radial)	1.000	0.923	0.667	0.667	0.752
SVM−CV:5 fold+Lasso−CV:5 fold (kernel: radial)	1.000	0.923	0.667	0.667	0.752
KNN+Lasso−CV:5 fold (k=5)	0.875	0.875	0.667	0.667	0.736
NN−MLP (cutoff:0.25, lr:0.001, bs:5, ep:50, dropout:0.25)	0.667	0.667	0.667	0.667	0.667
NN−MLP (cutoff:0.25, lr:0.001, bs:5, ep:50, dropout:0.5)	0.667	0.667	0.667	0.667	0.667
NN−MLP (cutoff:0.25, lr:0.001, bs:5, ep:50, dropout:0.75)	0.667	0.667	0.667	0.667	0.667
NN−MLP (cutoff:0.25, lr:0.005, bs:5, ep:50, dropout:0.25)	0.667	0.667	0.667	0.667	0.667
NN−MLP (cutoff:0.25, lr:0.005, bs:5, ep:50, dropout:0.5)	0.667	0.667	0.667	0.667	0.667
NN−MLP (cutoff:0.25, lr:0.005, bs:5, ep:50, dropout:0.75)	0.667	0.667	0.667	0.667	0.667
NN−MLP (cutoff:0.25, lr:0.01, bs:5, ep:50, dropout:0.25)	0.667	0.667	0.667	0.667	0.667
NN−MLP (cutoff:0.25, lr:0.01, bs:5, ep:50, dropout:0.5)	0.667	0.667	0.667	0.667	0.667
NN−MLP (cutoff:0.25, lr:0.01, bs:5, ep:50, dropout:0.75)	0.667	0.667	0.667	0.667	0.667
NN−MLP (cutoff:0.25, lr:0.05, bs:5, ep:50, dropout:0.25)	0.667	0.667	0.667	0.667	0.667
NN−MLP (cutoff:0.25, lr:0.05, bs:5, ep:50, dropout:0.5)	0.667	0.667	0.667	0.667	0.667
NN−MLP (cutoff:0.25, lr:0.05, bs:5, ep:50, dropout:0.75)	0.667	0.667	0.667	0.667	0.667
NaiveBayes	1.000	0.923	0.222	0.667	0.604
NN−MLP (cutoff:0.5, lr:0.005, bs:5, ep:50, dropout:0.25)	0.667	0.667	0.667	0.222	0.519
LR (cutoff:0.25)	1.000	0.444	0.000	1.000	0.481
LR (cutoff:0.5)	1.000	0.444	0.000	1.000	0.481
LR (cutoff:0.75)	1.000	0.444	0.000	1.000	0.481
Lasso.R−CV:5 fold (cutoff:0.25)	1.000	1.000	0.000	0.000	0.333
ENR−CV:5 fold (cutoff:0.25, alpha:0.2)	1.000	1.000	0.000	0.000	0.333
ENR−CV:5 fold (cutoff:0.25, alpha:0.3)	1.000	1.000	0.000	0.000	0.333
ENR−CV:5 fold (cutoff:0.25, alpha:0.4)	1.000	1.000	0.000	0.000	0.333
ENR−CV:5 fold (cutoff:0.25, alpha:0.5)	1.000	1.000	0.000	0.000	0.333
ENR−CV:5 fold (cutoff:0.25, alpha:0.6)	1.000	1.000	0.000	0.000	0.333
ENR−CV:5 fold (cutoff:0.25, alpha:0.7)	1.000	1.000	0.000	0.000	0.333
ENR−CV:5 fold (cutoff:0.5, alpha:0.7)	1.000	1.000	0.000	0.000	0.333
ENR−CV:5 fold (cutoff:0.25, alpha:0.8)	1.000	1.000	0.000	0.000	0.333
ENR−CV:5 fold (cutoff:0.5, alpha:0.8)	1.000	1.000	0.000	0.000	0.333
ENR−CV:5 fold (cutoff:0.25, alpha:0.9)	1.000	1.000	0.000	0.000	0.333

Average of test set (axis: 0, 0.2, 0.4, 0.6, 0.8)

F−score: 1, 0.5, 0

Data set: DatasetA (train set), DatasetB (test set), DatasetC (test set), DatasetD (test set)

S4

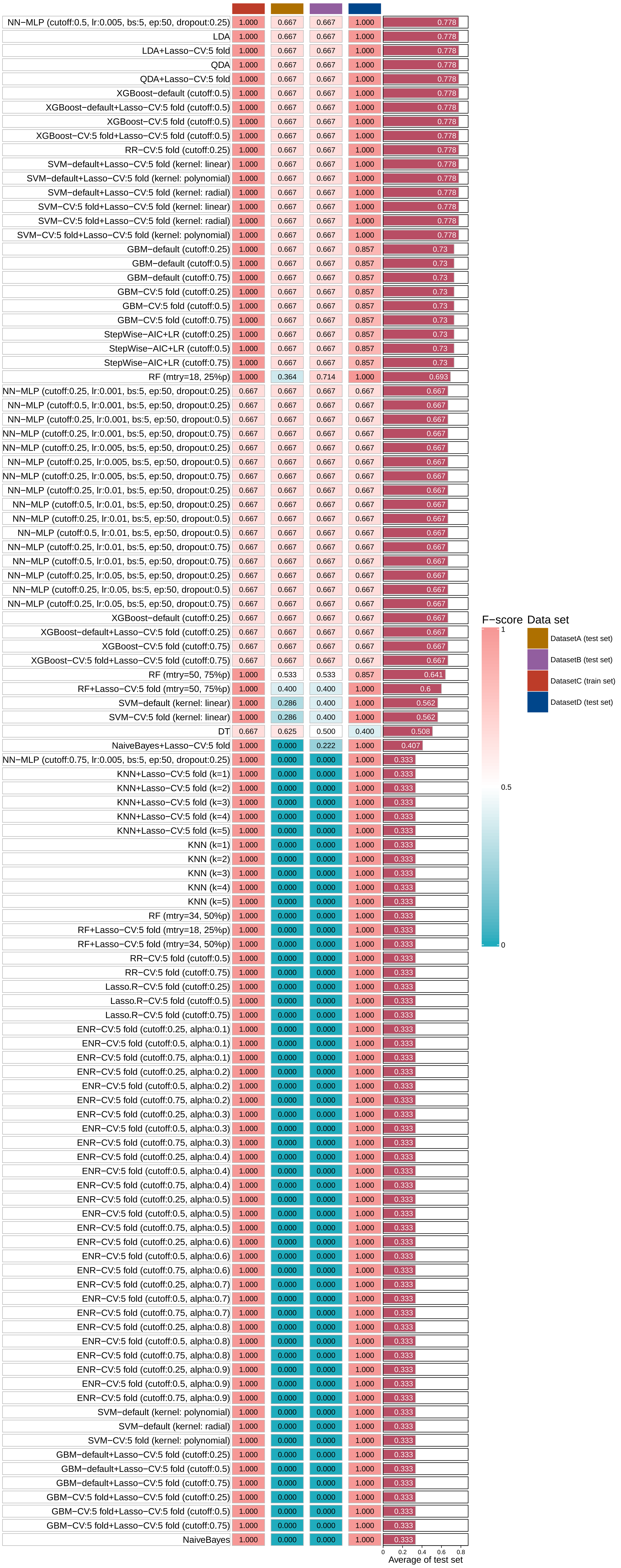

NN−MLP (cutoff:0.5, lr:0.005, bs:5, ep:50, dropout:0.25) 1.000 0.667 0.667 1.000 0.778
LDA 1.000 0.667 0.667 1.000 0.778
LDA+Lasso−CV:5 fold 1.000 0.667 0.667 1.000 0.778
QDA 1.000 0.667 0.667 1.000 0.778
QDA+Lasso−CV:5 fold 1.000 0.667 0.667 1.000 0.778
XGBoost−default (cutoff:0.5) 1.000 0.667 0.667 1.000 0.778
XGBoost−default+Lasso−CV:5 fold (cutoff:0.5) 1.000 0.667 0.667 1.000 0.778
XGBoost−CV:5 fold (cutoff:0.5) 1.000 0.667 0.667 1.000 0.778
XGBoost−CV:5 fold+Lasso−CV:5 fold (cutoff:0.5) 1.000 0.667 0.667 1.000 0.778
RR−CV:5 fold (cutoff:0.25) 1.000 0.667 0.667 1.000 0.778
SVM−default+Lasso−CV:5 fold (kernel: linear) 1.000 0.667 0.667 1.000 0.778
SVM−default+Lasso−CV:5 fold (kernel: polynomial) 1.000 0.667 0.667 1.000 0.778
SVM−default+Lasso−CV:5 fold (kernel: radial) 1.000 0.667 0.667 1.000 0.778
SVM−CV:5 fold+Lasso−CV:5 fold (kernel: linear) 1.000 0.667 0.667 1.000 0.778
SVM−CV:5 fold+Lasso−CV:5 fold (kernel: radial) 1.000 0.667 0.667 1.000 0.778
SVM−CV:5 fold+Lasso−CV:5 fold (kernel: polynomial) 1.000 0.667 0.667 1.000 0.778
GBM−default (cutoff:0.25) 1.000 0.667 0.667 0.857 0.73
GBM−default (cutoff:0.5) 1.000 0.667 0.667 0.857 0.73
GBM−default (cutoff:0.75) 1.000 0.667 0.667 0.857 0.73
GBM−CV:5 fold (cutoff:0.25) 1.000 0.667 0.667 0.857 0.73
GBM−CV:5 fold (cutoff:0.5) 1.000 0.667 0.667 0.857 0.73
GBM−CV:5 fold (cutoff:0.75) 1.000 0.667 0.667 0.857 0.73
StepWise−AIC+LR (cutoff:0.25) 1.000 0.667 0.667 0.857 0.73
StepWise−AIC+LR (cutoff:0.5) 1.000 0.667 0.667 0.857 0.73
StepWise−AIC+LR (cutoff:0.75) 1.000 0.667 0.667 0.857 0.73
RF (mtry=18, 25%p) 1.000 0.364 0.714 1.000 0.693
NN−MLP (cutoff:0.25, lr:0.001, bs:5, ep:50, dropout:0.25) 0.667 0.667 0.667 0.667 0.667
NN−MLP (cutoff:0.5, lr:0.001, bs:5, ep:50, dropout:0.25) 0.667 0.667 0.667 0.667 0.667
NN−MLP (cutoff:0.25, lr:0.001, bs:5, ep:50, dropout:0.5) 0.667 0.667 0.667 0.667 0.667
NN−MLP (cutoff:0.25, lr:0.001, bs:5, ep:50, dropout:0.75) 0.667 0.667 0.667 0.667 0.667
NN−MLP (cutoff:0.25, lr:0.005, bs:5, ep:50, dropout:0.25) 0.667 0.667 0.667 0.667 0.667
NN−MLP (cutoff:0.25, lr:0.005, bs:5, ep:50, dropout:0.5) 0.667 0.667 0.667 0.667 0.667
NN−MLP (cutoff:0.25, lr:0.005, bs:5, ep:50, dropout:0.75) 0.667 0.667 0.667 0.667 0.667
NN−MLP (cutoff:0.25, lr:0.01, bs:5, ep:50, dropout:0.25) 0.667 0.667 0.667 0.667 0.667
NN−MLP (cutoff:0.5, lr:0.01, bs:5, ep:50, dropout:0.25) 0.667 0.667 0.667 0.667 0.667
NN−MLP (cutoff:0.25, lr:0.01, bs:5, ep:50, dropout:0.5) 0.667 0.667 0.667 0.667 0.667
NN−MLP (cutoff:0.5, lr:0.01, bs:5, ep:50, dropout:0.5) 0.667 0.667 0.667 0.667 0.667
NN−MLP (cutoff:0.25, lr:0.01, bs:5, ep:50, dropout:0.75) 0.667 0.667 0.667 0.667 0.667
NN−MLP (cutoff:0.5, lr:0.01, bs:5, ep:50, dropout:0.75) 0.667 0.667 0.667 0.667 0.667
NN−MLP (cutoff:0.25, lr:0.05, bs:5, ep:50, dropout:0.25) 0.667 0.667 0.667 0.667 0.667
NN−MLP (cutoff:0.25, lr:0.05, bs:5, ep:50, dropout:0.5) 0.667 0.667 0.667 0.667 0.667
NN−MLP (cutoff:0.25, lr:0.05, bs:5, ep:50, dropout:0.75) 0.667 0.667 0.667 0.667 0.667
XGBoost−default (cutoff:0.25) 0.667 0.667 0.667 0.667 0.667
XGBoost−default+Lasso−CV:5 fold (cutoff:0.25) 0.667 0.667 0.667 0.667 0.667
XGBoost−CV:5 fold (cutoff:0.75) 0.667 0.667 0.667 0.667 0.667
XGBoost−CV:5 fold+Lasso−CV:5 fold (cutoff:0.75) 0.667 0.667 0.667 0.667 0.667
RF (mtry=50, 75%p) 1.000 0.533 0.533 0.857 0.641
RF+Lasso−CV:5 fold (mtry=50, 75%p) 1.000 0.400 0.400 1.000 0.6
SVM−default (kernel: linear) 1.000 0.286 0.400 1.000 0.562
SVM−CV:5 fold (kernel: linear) 1.000 0.286 0.400 1.000 0.562
DT 0.667 0.625 0.500 0.400 0.508
NaiveBayes+Lasso−CV:5 fold 1.000 0.000 0.222 1.000 0.407
NN−MLP (cutoff:0.75, lr:0.005, bs:5, ep:50, dropout:0.25) 1.000 0.000 0.000 1.000 0.333
KNN+Lasso−CV:5 fold (k=1) 1.000 0.000 0.000 1.000 0.333
KNN+Lasso−CV:5 fold (k=2) 1.000 0.000 0.000 1.000 0.333
KNN+Lasso−CV:5 fold (k=3) 1.000 0.000 0.000 1.000 0.333
KNN+Lasso−CV:5 fold (k=4) 1.000 0.000 0.000 1.000 0.333
KNN+Lasso−CV:5 fold (k=5) 1.000 0.000 0.000 1.000 0.333
KNN (k=1) 1.000 0.000 0.000 1.000 0.333
KNN (k=2) 1.000 0.000 0.000 1.000 0.333
KNN (k=3) 1.000 0.000 0.000 1.000 0.333
KNN (k=4) 1.000 0.000 0.000 1.000 0.333
KNN (k=5) 1.000 0.000 0.000 1.000 0.333
RF (mtry=34, 50%p) 1.000 0.000 0.000 1.000 0.333
RF+Lasso−CV:5 fold (mtry=18, 25%p) 1.000 0.000 0.000 1.000 0.333
RF+Lasso−CV:5 fold (mtry=34, 50%p) 1.000 0.000 0.000 1.000 0.333
RR−CV:5 fold (cutoff:0.5) 1.000 0.000 0.000 1.000 0.333
RR−CV:5 fold (cutoff:0.75) 1.000 0.000 0.000 1.000 0.333
Lasso.R−CV:5 fold (cutoff:0.25) 1.000 0.000 0.000 1.000 0.333
Lasso.R−CV:5 fold (cutoff:0.5) 1.000 0.000 0.000 1.000 0.333
Lasso.R−CV:5 fold (cutoff:0.75) 1.000 0.000 0.000 1.000 0.333
ENR−CV:5 fold (cutoff:0.25, alpha:0.1) 1.000 0.000 0.000 1.000 0.333
ENR−CV:5 fold (cutoff:0.5, alpha:0.1) 1.000 0.000 0.000 1.000 0.333
ENR−CV:5 fold (cutoff:0.75, alpha:0.1) 1.000 0.000 0.000 1.000 0.333
ENR−CV:5 fold (cutoff:0.25, alpha:0.2) 1.000 0.000 0.000 1.000 0.333
ENR−CV:5 fold (cutoff:0.5, alpha:0.2) 1.000 0.000 0.000 1.000 0.333
ENR−CV:5 fold (cutoff:0.75, alpha:0.2) 1.000 0.000 0.000 1.000 0.333
ENR−CV:5 fold (cutoff:0.25, alpha:0.3) 1.000 0.000 0.000 1.000 0.333
ENR−CV:5 fold (cutoff:0.5, alpha:0.3) 1.000 0.000 0.000 1.000 0.333
ENR−CV:5 fold (cutoff:0.75, alpha:0.3) 1.000 0.000 0.000 1.000 0.333
ENR−CV:5 fold (cutoff:0.25, alpha:0.4) 1.000 0.000 0.000 1.000 0.333
ENR−CV:5 fold (cutoff:0.5, alpha:0.4) 1.000 0.000 0.000 1.000 0.333
ENR−CV:5 fold (cutoff:0.75, alpha:0.4) 1.000 0.000 0.000 1.000 0.333
ENR−CV:5 fold (cutoff:0.25, alpha:0.5) 1.000 0.000 0.000 1.000 0.333
ENR−CV:5 fold (cutoff:0.5, alpha:0.5) 1.000 0.000 0.000 1.000 0.333
ENR−CV:5 fold (cutoff:0.75, alpha:0.5) 1.000 0.000 0.000 1.000 0.333
ENR−CV:5 fold (cutoff:0.25, alpha:0.6) 1.000 0.000 0.000 1.000 0.333
ENR−CV:5 fold (cutoff:0.5, alpha:0.6) 1.000 0.000 0.000 1.000 0.333
ENR−CV:5 fold (cutoff:0.75, alpha:0.6) 1.000 0.000 0.000 1.000 0.333
ENR−CV:5 fold (cutoff:0.25, alpha:0.7) 1.000 0.000 0.000 1.000 0.333
ENR−CV:5 fold (cutoff:0.5, alpha:0.7) 1.000 0.000 0.000 1.000 0.333
ENR−CV:5 fold (cutoff:0.75, alpha:0.7) 1.000 0.000 0.000 1.000 0.333
ENR−CV:5 fold (cutoff:0.25, alpha:0.8) 1.000 0.000 0.000 1.000 0.333
ENR−CV:5 fold (cutoff:0.5, alpha:0.8) 1.000 0.000 0.000 1.000 0.333
ENR−CV:5 fold (cutoff:0.75, alpha:0.8) 1.000 0.000 0.000 1.000 0.333
ENR−CV:5 fold (cutoff:0.25, alpha:0.9) 1.000 0.000 0.000 1.000 0.333
ENR−CV:5 fold (cutoff:0.5, alpha:0.9) 1.000 0.000 0.000 1.000 0.333
ENR−CV:5 fold (cutoff:0.75, alpha:0.9) 1.000 0.000 0.000 1.000 0.333
SVM−default (kernel: polynomial) 1.000 0.000 0.000 1.000 0.333
SVM−default (kernel: radial) 1.000 0.000 0.000 1.000 0.333
SVM−CV:5 fold (kernel: polynomial) 1.000 0.000 0.000 1.000 0.333
GBM−default+Lasso−CV:5 fold (cutoff:0.25) 1.000 0.000 0.000 1.000 0.333
GBM−default+Lasso−CV:5 fold (cutoff:0.5) 1.000 0.000 0.000 1.000 0.333
GBM−default+Lasso−CV:5 fold (cutoff:0.75) 1.000 0.000 0.000 1.000 0.333
GBM−CV:5 fold+Lasso−CV:5 fold (cutoff:0.25) 1.000 0.000 0.000 1.000 0.333
GBM−CV:5 fold+Lasso−CV:5 fold (cutoff:0.5) 1.000 0.000 0.000 1.000 0.333
GBM−CV:5 fold+Lasso−CV:5 fold (cutoff:0.75) 1.000 0.000 0.000 1.000 0.333
NaiveBayes 1.000 0.000 0.000 1.000 0.333
0 0.2 0.4 0.6 0.8
Average of test set
F−score
1
0.5
0
Data set
DatasetA (test set)
DatasetB (test set)
DatasetC (train set)
DatasetD (test set)
