# Supplementary material for: Multi-source data integration and ensemble machine learning analysis of the role of Corilagin in regulating endoplasmic reticulum stress and alleviating intestinal ischemia reperfusion injury in mice
Source: Front Pharmacol. 2026 Oct 9;17:1966534. doi: 10.3389/fphar.2026.1966534 (PMC13691303; doi:10.3389/fphar.2026.1966534)
Supplement: Supplementary file 2 [file Supplementaryfile7.pdf]

Adjusted P values for the 12 selected core genes

IIRI			
gene	logFC	P.Value	adj.P.Val
IL10	1.875856872	8.612E-08	1.795E-05
ABL2	1.784949984	1.666E-05	6.313E-04
SELE	2.501684167	1.070E-07	2.106E-05
FGF2	1.778938833	6.571E-08	1.474E-05
GEM	3.577117688	7.410E-09	2.848E-06
PRKCZ	-1.163225729	2.913E-05	9.369E-04
ABCG2	-1.091104063	6.440E-06	3.387E-04
C5AR1	1.427532354	6.855E-06	3.536E-04
AXL	1.472750646	1.053E-06	1.030E-04
HMOX1	4.751297583	1.392E-11	2.858E-08
BDKRB1	2.022982786	2.295E-07	3.563E-05
ABCC1	1.14685476	2.972E-07	4.443E-05
