# Supplementary material for: Multi-source data integration and ensemble machine learning analysis of the role of Corilagin in regulating endoplasmic reticulum stress and alleviating intestinal ischemia reperfusion injury in mice
Source: Front Pharmacol. 2026 Oct 9;17:1966534. doi: 10.3389/fphar.2026.1966534 (PMC13691303; doi:10.3389/fphar.2026.1966534)
Supplement: Supplementary file 3 [file Supplementaryfile8.pdf]

Method: Cellular thermal shift assay for evaluating Corilagin – NRF2 target engagement in IEC-6 cells

A cellular thermal shift assay (CETSA) was performed to evaluate the intracellular target engagement between Corilagin and NRF2 in IEC-6 rat small intestinal crypt epithelial cells. IEC-6 cells were cultured and treated with Corilagin or the corresponding vehicle control under the conditions described above. After treatment, the cells were harvested, washed twice with ice-cold phosphate-buffered saline (PBS), and resuspended in PBS supplemented with a protease inhibitor cocktail. Equal volumes of the cell suspension were divided into four aliquots and heated at 45, 50, 55, or 60 °C for 3 min using a thermal cycler, followed by cooling at room temperature for 3 min. The heated cells were subsequently lysed by three freeze – thaw cycles using liquid nitrogen and a 37 °C water bath. Cell lysates were centrifuged at 20,000 × g for 20 min at 4 °C to remove heat-induced protein aggregates and cellular debris, and the supernatants containing the soluble protein fractions were carefully collected. Equivalent volumes of the soluble fractions were mixed with SDS loading buffer and subjected to SDS – polyacrylamide gel electrophoresis and Western blotting . NRF2 protein levels were detected using primary antibody of NRF2 (Proteintech, China, 33123-1-AP, 1:1000) , and band intensities were quantified using ImageJ. For each treatment condition, the soluble NRF2 signal at 45 °C was defined as 100%, and the relative abundance of soluble NRF2 at 50, 55, and 60 °C was calculated accordingly to generate thermal stability profiles. Three independent biological experiments were performed.

Result: Corilagin increased the thermal stability of NRF2 in IEC-6 cells

To further evaluate the predicted Corilagin – NRF2 interaction at the cellular level, a cellular thermal shift assay (CETSA) was performed in IEC-6 cells. With increasing temperature, the amount of soluble NRF2 progressively decreased in vehicle-treated cells. In contrast, Corilagin-treated cells retained higher levels of NRF2 at elevated temperatures, with the difference becoming more apparent at 55 and 60 °C. β-actin levels remained relatively stable across the temperature gradient. These findings indicate that Corilagin increased the thermal stability of NRF2 in IEC-6 cells, providing cellular target-engagement evidence consistent with the predicted Corilagin – NRF2 interaction.

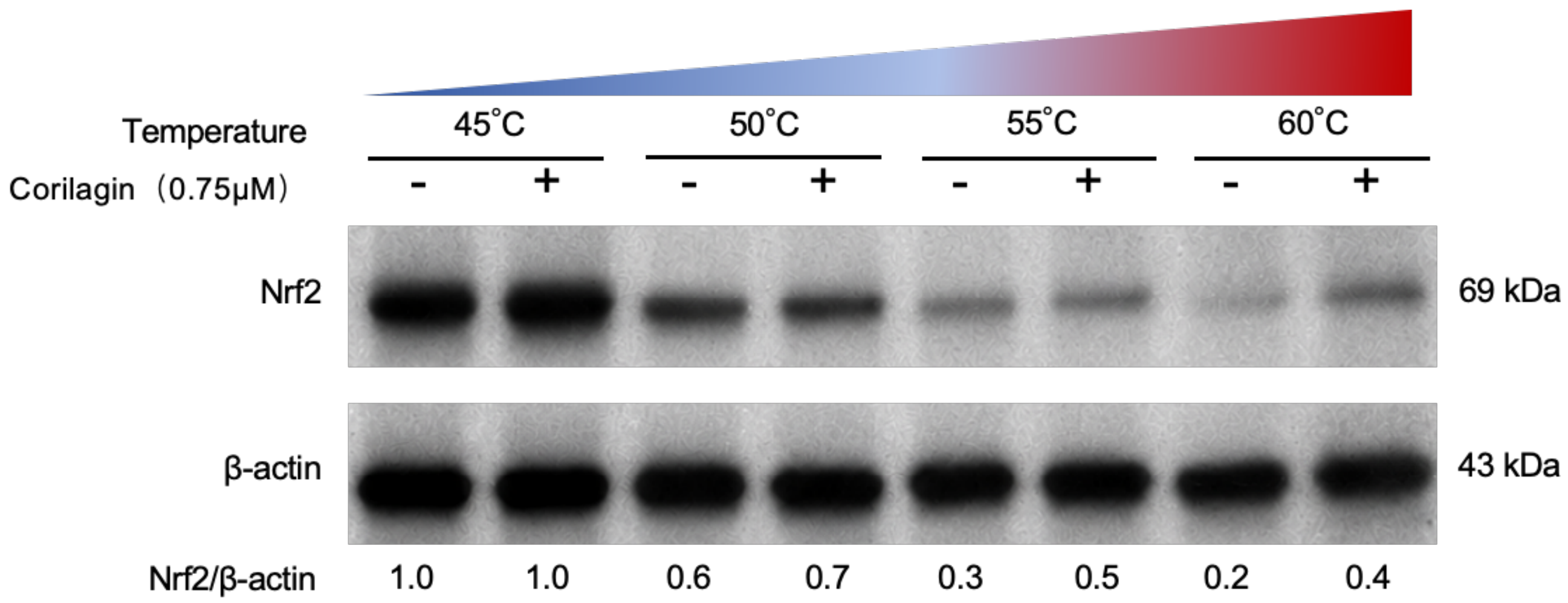

Supplementary Figure S7. Cellular thermal shift assay analysis of the Corilagin – NRF2 interaction in IEC-6 cells.

Representative Western blot images of NRF2 and β-actin in IEC-6 cells treated with vehicle or Corilagin and subsequently exposed to 45, 50, 55, or 60 °C. NRF2 abundance progressively decreased with increasing temperature in vehicle-treated cells, whereas Corilagin treatment increased the retention of soluble NRF2, particularly at higher temperatures. β-actin was used as the loading control. These results support increased thermal stability of NRF2 following Corilagin treatment and are consistent with intracellular target engagement between Corilagin and NRF2.
